# Molecular level detection and localization of mechanical damage in collagen enabled by collagen hybridizing peptides

**DOI:** 10.1038/ncomms14913

**Published:** 2017-03-22

**Authors:** Jared L. Zitnay, Yang Li, Zhao Qin, Boi Hoa San, Baptiste Depalle, Shawn P. Reese, Markus J. Buehler, S. Michael Yu, Jeffrey A. Weiss

**Affiliations:** 1 Department of Bioengineering, University of Utah, Salt Lake City, Utah 84112, USA; 2 Scientific Computing and Imaging Institute, University of Utah, Salt Lake City, Utah 84112, USA; 3 Laboratory for Atomistic and Molecular Mechanics, Department of Civil and Environmental Engineering, Massachusetts Institute of Technology, Cambridge, Massachusetts 02139, USA; 4 Department of Pharmaceutics and Pharmaceutical Chemistry, University of Utah, Salt Lake City, Utah 84112, USA; 5 Department of Orthopedics, University of Utah, Salt Lake City, Utah 84108, USA

## Abstract

Mechanical injury to connective tissue causes changes in collagen structure and material behaviour, but the role and mechanisms of molecular damage have not been established. In the case of mechanical subfailure damage, no apparent macroscale damage can be detected, yet this damage initiates and potentiates in pathological processes. Here, we utilize collagen hybridizing peptide (CHP), which binds unfolded collagen by triple helix formation, to detect molecular level subfailure damage to collagen in mechanically stretched rat tail tendon fascicle. Our results directly reveal that collagen triple helix unfolding occurs during tensile loading of collagenous tissues and thus is an important damage mechanism. Steered molecular dynamics simulations suggest that a likely mechanism for triple helix unfolding is intermolecular shearing of collagen α-chains. Our results elucidate a probable molecular failure mechanism associated with subfailure injuries, and demonstrate the potential of CHP targeting for diagnosis, treatment and monitoring of tissue disease and injury.

## Abstract (web summary)

Collagen denaturation is thought to occur during tissue mechanical damage, but its role in damage initiation is still unclear. Here, the authors use a collagen hybridizing peptide to provide insights into the molecular mechanisms leading to collagen unfolding during tendon mechanical stretch.

Many painful and physically debilitating conditions involve subfailure tissue damage, or mechanical damage to seemingly intact connective tissues. Examples include rotator cuff disease^1^,^2^, tendinosis^3^,^4^, whiplash^5^,^6^ and low back pain^6^,^7^ which result from overuse, repeated subfailure injury, or trauma. As collagen is the major structural component of connective tissues, these injuries presumably involve damage to the hierarchical structure of collagen at one or more physical scales. Previous studies have documented mechanical damage to connective tissues following subfailure loading^8^,^9^,^10^,^11^,^12^,^13^,^14^, and demonstrated that this damage is linked to clinically relevant pathological symptoms. However, the exact nature and evolution of damage to collagen across its hierarchy of structure is poorly understood.

Several experimental^15^,^16^,^17^ and computational^18^,^19^ studies have indicated that molecular level damage to collagen (that is, collagen denaturation) occurs during overloading. For example, collagen fibrils in ruptured^20^ or repeatedly overloaded tendons are more susceptible to proteolytic digestion by trypsin than collagen from unloaded tendons^15^,^16^,^17^,^21^. Since trypsin can efficiently digest non-triple-helical structure, this was considered a sign that mechanical overload may have caused unfolding of the triple-helical collagen molecules. However, trypsin has been also reported to digest triple-helical collagen molecules by targeting their thermally unstable domain, if the collagen molecules are not assembled into the fibril structure^22^,^23^. Therefore it is unclear whether the increased susceptibility to trypsin digestion in overloaded tendons was the result of damage to collagen molecules or to collagen fibrils^15^,^16^,^17^,^21^, although additional experiments involving differential scanning calorimetry and low temperature digestion seem to suggest molecular damage in some instances^17^.

To date, studies that examined mechanical damage to collagen fibrils in tendon were performed on severely damaged samples (ruptured, or with repeated sub-rupture overloading)^15^,^16^,^17^,^20^,^21^,^24^, and it is unclear whether collagen denaturation plays a role in initiation of damage or it is merely a secondary outcome of the failure at the fibril, fiber and/or tissue levels. There is no direct and conclusive experimental evidence to indicate the extent or location of molecular level failure of collagen during moderate subfailure loading, or the mechanism of failure at the molecular level. This is mainly due to the lack of appropriate tools to directly probe minute structural changes in collagen molecules that may occur during subfailure loading.

A quantitative technique capable of direct detection and spatial localization of collagen molecular damage at multiple physical scales would be highly useful for understanding the relationship between tissue injury and molecular damage, while potentially providing a means for early detection and treatment of tissue injuries. This led us to consider the use of collagen hybridizing peptide (CHP), also known as collagen mimetic peptide, to detect molecular level damage to collagen^25^. CHP is a small synthetic peptide that mimics the triple-helical molecular structure of natural collagens and was demonstrated to hybridize with globally unfolded collagen strands^25^,^26^. CHP is comprised of glycine(G)–proline(P)–hydroxyproline(O) amino acid repeats and can form a stable homotrimeric triple helix virtually identical to that of collagen through hydrogen-bonding between strand backbones^25^. Traditionally, these triple-helical peptides have been used as a model system to study the structure and folding behaviour of collagens, and for engineered collagen mimetic biomaterials^27^,^28^,^29^. The GPO unit has the highest folding propensity for the triple-helical structure among all GXY triplets in a collagen sequence, and a peptide made of GPO repeats can form a more stable triple helix than natural collagen^29^. Previously, we reported that single-strand CHP with the sequence (GPO)_*n*_ can hybridize to unfolded collagen strands that have been denatured by heat or by protease activity, both *in vitro* and *in vivo*^26^. We demonstrated that CHP has negligible affinity to intact collagen and virtually no non-specific binding in tissues^26^ due to its neutral and hydrophilic sequence. Furthermore, a scrambled sequence CHP lacking triple-helical folding propensity showed negligible affinity to denatured collagen^26^,^30^, confirming that CHP binding in tissues is a direct result of triple-helical hybridization with unfolded collagen strands.

Considering the ability of CHP to specifically hybridize with the unfolded collagen triple helix, we hypothesized that carboxyfluorescein (CF) labeled CHP (CF-CHP) could be used to detect, localize and quantitatively compare molecular level damage to collagen caused by mechanical loading. We used CF-CHP to detect damage due to collagen unfolding during subfailure loading for a series of mechanically stretched rat tail tendon (RTT) fascicles. We show that the triple helix-mediated hybridization process effectively reports the location and level of molecular damage of the collagens in tendon, which provides new insights into the mechanism of mechanical damage to fibrous collagens in load bearing tissues. We also conducted steered molecular dynamics (MD) simulations to investigate the molecular response of the collagen triple helix under loading at the atomic scale and identified a new molecular mechanism that has been previously overlooked but is capable of causing permanent mechanical damage to fibrous collagens in load bearing tissues.

## Results

### Uniaxial tensile testing and CF-CHP binding

**Figure 1:**
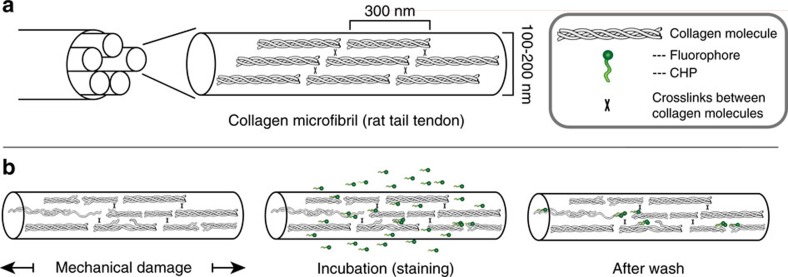
Collagen mechanical damage and CHP binding. (**a**) Simple schematic of triple-helical collagen organization in intact tendon microfibrils based on the Hodge–Petruska model^70^. (**b**) Intact RTT fascicles were stretched in uniaxial tension to initiate mechanical damage within the tissue structure, followed by incubation with single-strand CF-CHP to allow triple helix-mediated hybridization to unfolded collagen strands, and washing to remove unbound CHP.

**Figure 2:**
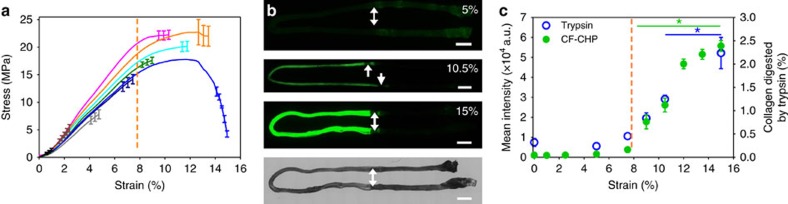
Fluorescence intensity and stress in tail tendon fascicles at incremental strains. (**a**) Average stress–strain curves for each of the strain groups tested (*n*=3 each curve). Fascicle mechanical behaviour encompassed the range of linear behaviour to tissue failure. For clarity, the standard error bars are only shown for the last 1% strain of each curve. (**b**) Representative whole-sample fluorescence scans of 5, 10.5 and 15% strain samples, and the corresponding brightfield image for the 15% strain sample, clearly showing an increase in CF-CHP staining with increased strain. In these images, fascicles have been folded on microscope slides showing the stretched section on the left half of the image and the clamped ends on the right half of the image. Arrows indicate the approximate location of clamping. Scale bars, 2 mm. (**c**) Mean pixel intensity (*n*=5, 0% strain; *n*=3, 1–15% strain, mean±s.e.m.) quantified from fluorescence images of loaded fascicles (left axis) and the average percentage of collagen digested by trypsin (right axis) from fascicles in each group (0, 5, 7.5, 9, 10.5, 15% strain, *n*=5, mean±s.e.m.). The orange dotted lines in **a** and **c** indicate the approximate transition strain from the linear region of the stress–strain curve to the onset of damage as identified by the deviation of the stress–strain curve from linearity, which correlates with onset of CF-CHP intensity.

We exploited the ability of CHP to target denatured collagen for the detection of molecular level mechanical damage to collagen in RTT under uniaxial extension (Fig. 1). Using a custom micro-mechanical test system (Supplementary Fig. 1a,b), RTT fascicles were subjected to a range of incremental strains up to complete failure (Fig. 2a). After mechanical loading and unloading at room temperature, each fascicle was incubated at 4 °C in a solution of CF-CHP [CF–G_3_–(GPO)_9_], washed and imaged using wide-field fluorescence microscopy to assess the CHP binding (Supplementary Fig. 1c). Measured CF-CHP fluorescence intensity increased with applied strain (Fig. 2b) and all strain groups above 7.5% exhibited significantly greater intensity than the unloaded control (Fig. 2c). There was little fluorescence intensity in the low-strain groups, with the initial intensity increase detected in 9% strain samples, followed by a pseudo-sigmoidal increase in fluorescence intensity as a function of strain (Fig. 2c). Comparison of mechanical testing and fluorescence intensity data (Fig. 2a,c) revealed that the onset of significant CHP binding, which took place between 7.5 and 9% strain, corresponded to a decrease in material tangent modulus of individual samples (marked by dotted orange line). The fluorescence intensity reached a plateau at a range of strain that corresponded to the end of the stress plateau and ultimate failure of the tissues (12–15%). These changes in tissue behaviour were also observed by plotting the average modulus as a function of strain (Supplementary Fig. 2a). The modulus reached a peak near 2.5% strain and this peak was significantly greater than the modulus at any of the other strain levels tested. The reduction in modulus following the peak at 2.5% strain demonstrates softening that was observed in the stress–strain curves (Fig. 2a), and the greatest decrease in modulus began between 6 and 8% strain. In the field of tissue biomechanics, it is well established that the transition from linear region to plateau region in the stress–strain curve (marked by dotted orange lines in Fig. 2a,c, decreasing modulus starting at 6–8% strain in Supplementary Fig. 2a) signifies permanent tissue damage^10^,^11^,^31^,^32^.

The direct correlation between this transition and the onset of CF-CHP binding, as well as the steady increase in CF-CHP binding over the plateau region of the stress–strain curve, suggest that unfolding of collagen molecules corresponds with the initiation and progression of mechanical damage to tendon during uniaxial stretching. To our knowledge, this was the first time a result suggested that collagen unfolding is taking place during subfailure loading, and therefore we attempted to verify it by another method. We modified the extraction method (trypsin digestion followed by hydroxyproline assay), which was previously used to detect denatured collagen for only highly damaged tendon samples^15^,^17^, and applied it to a series of incrementally strained RTT fascicles. After significant improvement of the original protocol (Supplementary Methods and Supplementary Fig. 3), we were able to detect small quantities of digested collagens, which correlated closely with the level of CHP binding (Fig. 2c). Strain groups above 9% strain exhibited a statistically significant increase in digested collagen compared to the unloaded controls. This result supports the outcome of the CF-CHP binding experiment that molecular level collagen denaturation is occurring even at low subfailure loading.

### Localization of CF-CHP binding using multiphoton imaging

**Figure 3:**
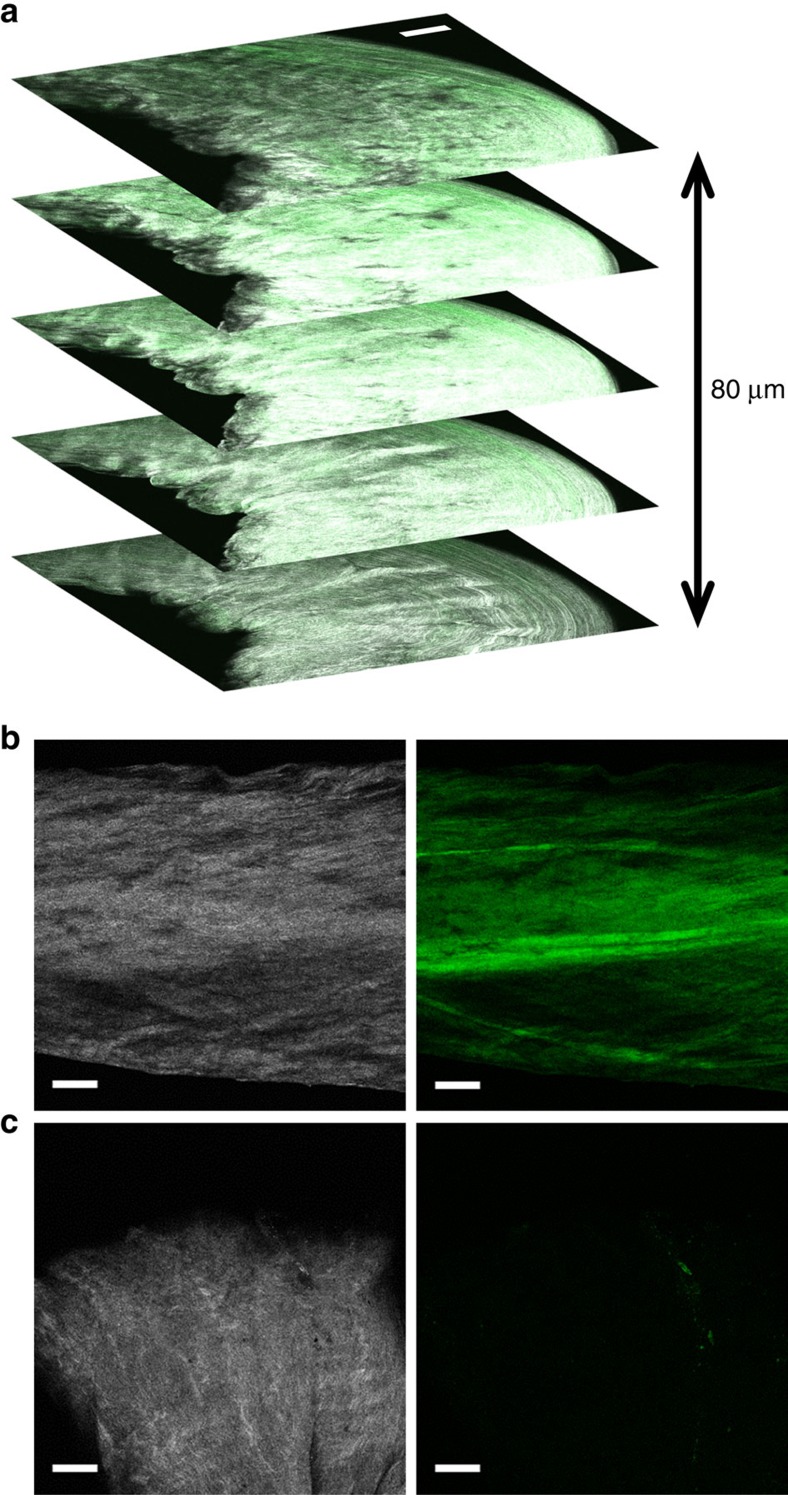
Multiphoton fluorescence localization of CF-CHP in mechanically damaged fascicles. Multiphoton images of collagen SHG (white) and CF two-photon excited fluorescence (TPEF, green). (**a**) Multiphoton z-stack combining both SHG and CF TPEF through the imaging depth for a single fascicle stretched to 7.5% strain. The presence of CF fluorescence through the 80 μm depth confirmed that CHP binding was not limited to the tissue surface. SHG and CF TPEF signals are separated in panels **b** and **c**. (**b**) Banded CHP staining pattern in a 15% strain sample revealed inhomogeneity in molecular level collagen damage. This pattern was prevalent in samples across the entire range of strains tested. (**c**) Images of the end of a sample that was cut using sharp scissors. Note the minimal CHP staining. Scale bars 100 μm for all panels.

CF-CHP fluorescence within fascicles was imaged using multiphoton confocal microscopy (Fig. 3) to correlate CF-CHP binding with tissue structure. A small group of the mechanically loaded fascicles were selected and imaged using a single excitation wavelength, acquiring the collagen second harmonic generation (SHG) signal (shown in white) on one channel, and the CF-CHP two-photon excited fluorescence signal (shown in green) on another channel. This multiphoton localization imaging allowed direct comparison of collagen organization with CF-CHP staining, which revealed staining patterns that were not evident in the wide-field fluorescence images. Z-stack images through the available imaging depth (∼100 μm) confirmed that CF-CHP had penetrated throughout the fascicles and was not limited to surface staining (Fig. 3a). Side-by-side comparison of the collagen (SHG) and CF-CHP signals in z-plane images revealed heterogeneous CF-CHP staining across the tested areas (Fig. 3b), thus indicating spatial heterogeneity of collagen molecule unfolding throughout the tissue. The heterogeneous staining often exhibited a banded appearance both at high strains (15% strain, Fig. 3b) and lower strains (7.5 and 13.5% strains, Supplementary Fig. 4), with the bands of increased fluorescence intensity oriented generally along the long axis of the fascicle. Some regions of increased CF-CHP signal co-localized with regions of strong SHG signal, while in other regions the increased CF-CHP signal co-localized with regions of weak or absent SHG signal. These observations of staining heterogeneity are in agreement with previous reports of inhomogeneous loading throughout the collagen hierarchy in ligaments and tendons during uniaxial testing^8^,^33^. There was minimal fluorescence signal in regions of the fascicles that were gripped by the mechanical testing clamps and in areas near the sample ends that were cut with a sharp blade during sample preparation (Fig. 3c). This was a surprising observation, since we expected that clamping and sharp cutting would result in fragmentation of the collagen molecule similar to proteolytic cleavage^26^,^34^. The absence of CF-CHP binding suggests that clamping and cutting do not involve tensile overload of the collagen molecules and that the CHP binding detects only tissue damage relevant to physiological mechanisms.

### TEM of CHP-nanoparticles bound to damaged collagen fibrils

**Figure 4:**
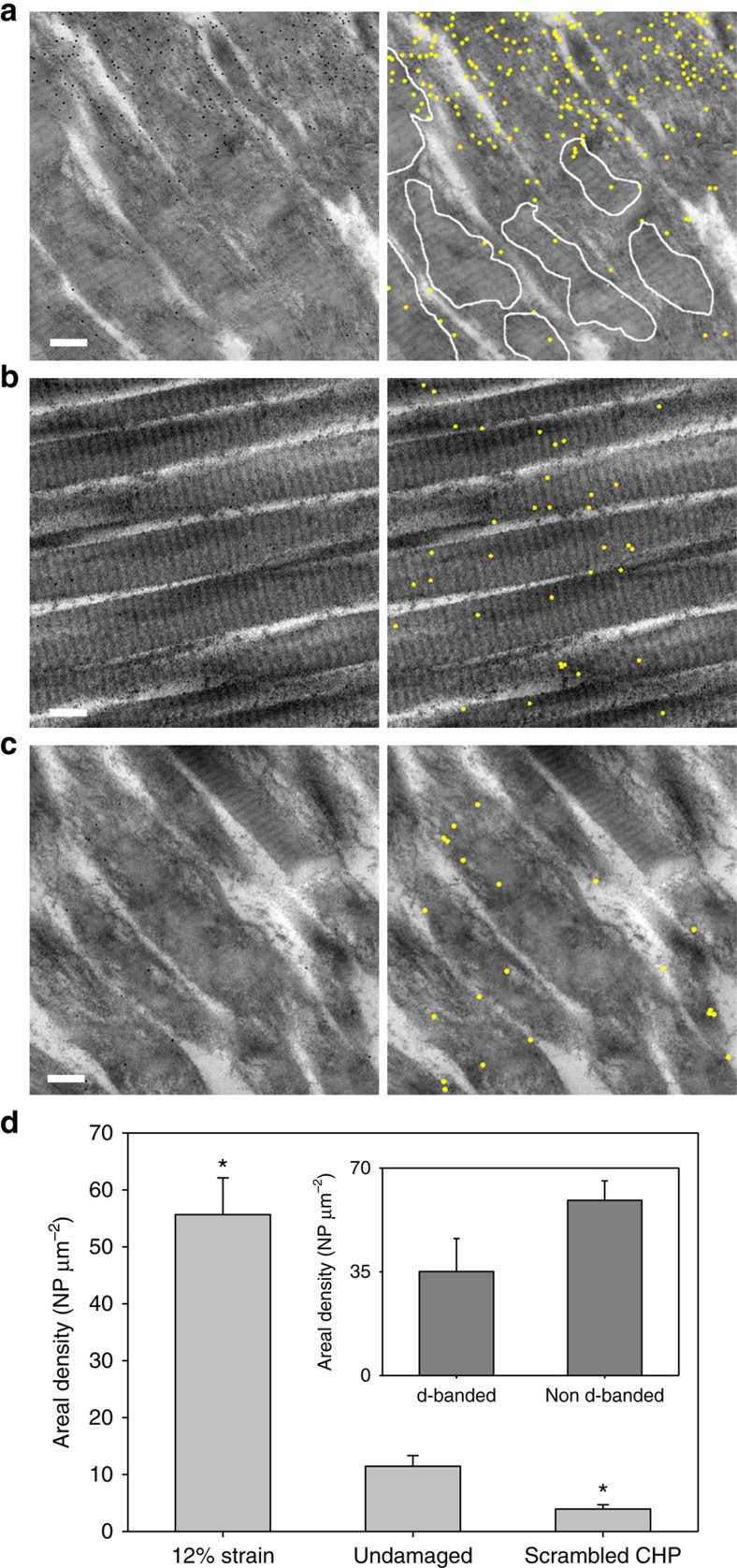
Transmission electron micrographs of NP-CHP binding to damaged collagen fibrils. Raw images are in the left column, while corresponding annotated images are in the right column. Yellow dots represent the identified NP-CHPs. (**a**) Damaged tissue from a 12% strain fascicle exhibited a high density of NP-CHP binding and a large amount of visible fibril disruption (that is, loss of d-banding pattern). The few areas with remaining d-banding pattern are outlined in white lines. (**b**) Undamaged tendon fascicle incubated with NP-CHP. (**c**) Damaged tissue from a 12% strain fascicle incubated with scrambled sequence NP-^S^CHP, showing low levels of non-specific binding. (**d**) Overall NP-CHP densities averaged from 5 randomly selected images from each group (mean±s.e.m.). Inset—Number of NP-CHP per μm^2^ inside and outside the areas with preserved d-banding (outlined areas in panel a) in the images of 12% strain fascicles (mean±s.e.m.). *indicates statistical significance from the undamaged group with *P*<0.05. Scale bars, 200 nm.

To correlate the mechanical unfolding of collagen molecules with the disruption of collagen fibrils, we visualized CHP hybridized denatured collagen using CHP-conjugated gold nanoparticles (NP-CHP) under transmission electron microscopy (TEM). TEM has long been used to interrogate the integrity of collagen fibrils through visualization of the characteristic 67 nm periodic d-banding pattern^35^. Previously using TEM, we demonstrated that NP-CHP binds to thermally denatured collagen molecules by triple-helical hybridization^36^. An unloaded fascicle and a fascicle stretched to 12% strain were treated with NP-CHP, washed and imaged by TEM. NP-CHP binding density was noticeably higher in the loaded fascicle (Fig. 4a), while only a low level of binding was seen in the undamaged fascicle images (Fig. 4b). An even lower number of NPs were found in the images of the damaged fascicle probed by gold nanoparticles conjugated with a scrambled CHP sequence (NP-^S^CHP, Fig. 4c). It was noted that the 12% strain fascicle had extensive visible fibril disruption, evidenced by an absence of the characteristic d-banding pattern and few remaining regions with d-banding (Fig. 4a, white outline), which appeared blurrier than d-banding from the intact fascicles (Fig. 4b). Five randomly chosen images on each fascicle (Supplementary Figs 5–7) were analyzed for NP quantification (Fig. 4d). The mean areal density of NP on the 12% strain fascicle was remarkably low for the sequence-scrambled ^S^CHP, confirming our observation that CHP binding in damaged fascicles is specifically via the triple-helical hybridization to unfolded collagen. The mean areal density of NP-CHP in the 12% strain group was significantly higher than the undamaged group (*P*<0.05). The mean areal density of NP-CHP binding in regions with visible collagen fibril disruption was higher than the mean areal density in regions with retained d-banding in the 12% strain samples, although the difference was not statistically significant (*P*=0.10, Fig. 4d inset).

### Cyclic tensile testing and CF-CHP binding

**Figure 5:**
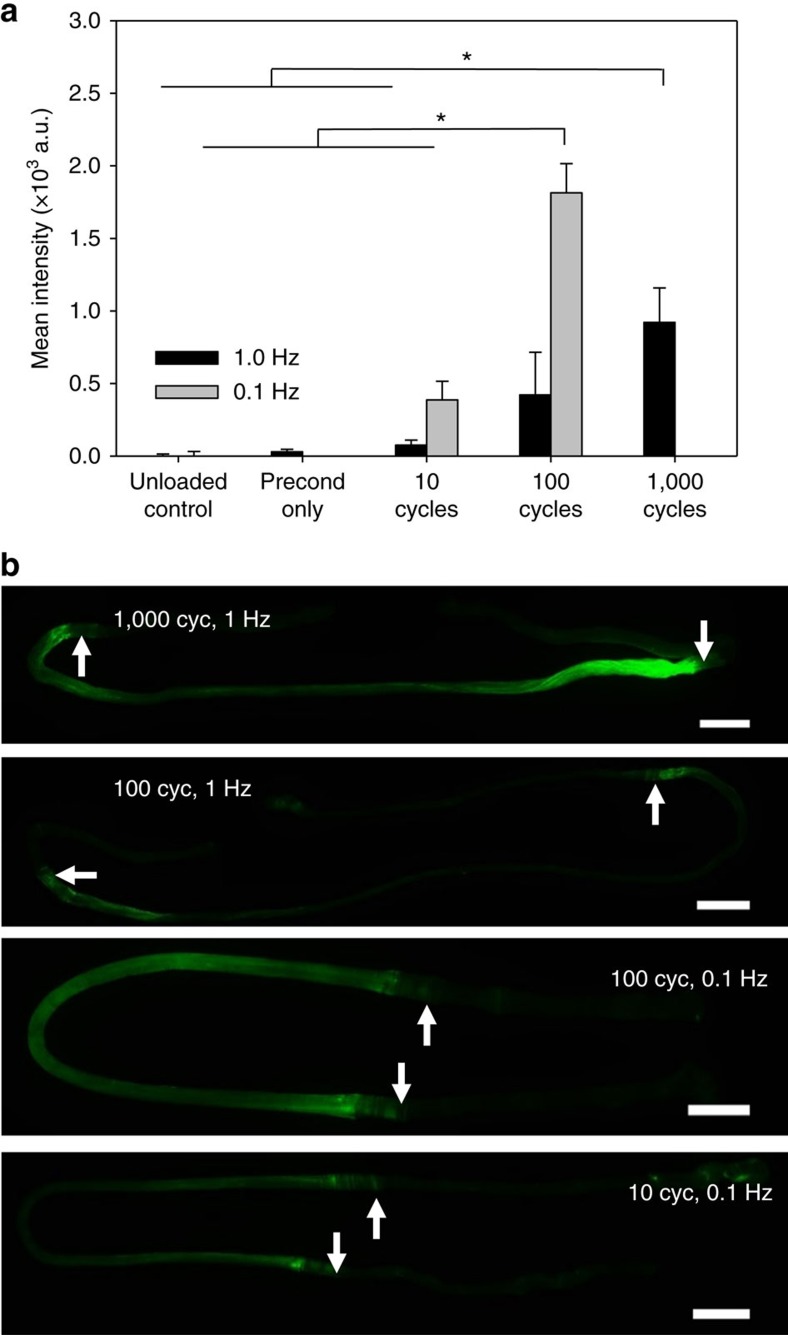
CF-CHP binding to fatigue loaded fascicles. (**a**) Mean fluorescence intensity versus fatigue loading condition for samples loaded between 1 and 5% strain at 0.1 and 1.0 Hz (*n*=3, mean±s.e.m.). Statistically significant intensity increase from the controls was observed in 0.1 Hz samples after 100 cycles and in 1.0 Hz samples after 1,000 cycles. (**b**) Representative wide-field fluorescence images reveal different CHP staining patterns between the 0.1 and 1.0 Hz samples. The staining images help explain the apparent overall high intensity of 0.1 Hz samples, as CHP was bound to a larger area in 0.1 Hz samples compared to 1.0 Hz samples tested for the same number of cycles. Arrows indicate approximate location of clamps. Scale bars, 2 mm.

To investigate the effects of repeated subfailure loading on collagen molecular damage, we tested fascicles in cyclic fatigue at physiological and quasi-static loading frequencies^37^. Fascicles were subjected to repeated sinusoidal strain cycles between 1 and 5% tensile strain. The strain level of 5% represents approximately half of the failure strain, yet it did not produce damage as measured by CF-CHP staining in the single stretch experiments described above (Fig. 2). Fascicles were strained at 1.0 Hz for either 10, 100 or 1000 cycles, or at 0.1 Hz for either 10 or 100 cycles. These two loading frequencies were chosen to represent two relevant cases of viscoelastic tissue behaviour, with 1 Hz loading simulating a physiologically relevant cyclic load and 0.1 Hz loading simulating a repeated quasi-static load. The number of loading cycles had a significant effect on fluorescence intensity; fascicles loaded for 100 cycles at 0.1 Hz or 1,000 cycles at 1.0 Hz exhibited a statistically significant increase in CF-CHP staining compared to the unloaded controls (Fig. 5a). This increase in CF-CHP staining intensity correlated with changes in material behaviour at the fascicle level, where the stress borne by the tissue as well as the linear modulus during loading decreased with increasing number of loading cycles (Supplementary Fig. 2b–d).

The two different loading frequencies produced different CF-CHP staining patterns (Fig. 5b). While samples tested at 1 Hz exhibited staining concentrated at the area close to clamped regions of the fascicle (Fig. 5b, arrows), samples tested at 0.1 Hz exhibited a homogeneous staining pattern over the length of the sample, similar to that observed for the tensile test specimens. This behaviour may be explained by the biphasic nature of ligaments and tendons, consisting of a solid matrix of primarily collagen and a fluid phase. Under tensile loading, transient pressures develop within the tissue resulting in fluid flux^38^. This affects stress distribution between solid and fluid phases, as demonstrated experimentally for articular cartilage^39^, and imparts rate-dependent material behaviour^40^. The 1.0 Hz cyclic strain occurs over a timescale that is much shorter than that required for fluid exudation^41^,^42^, causing the tissue to remain unequilibrated and the fluid phase to bear a substantial portion of the tissue stress. The 0.1 Hz loading rate allowed fluid depressurization, exposing the collagen phase of the tissue to higher stresses. The presence of collagen damage after repeated cyclic loading to 5% strain, along with the localized CHP binding observed in multiphoton imaging of low-strain samples, suggests that a certain level of collagen molecular damage in connective tissues may be a normal part of tissue function during physiological activity.

### Simulation and mechanism of collagen molecule unfolding

**Figure 6:**
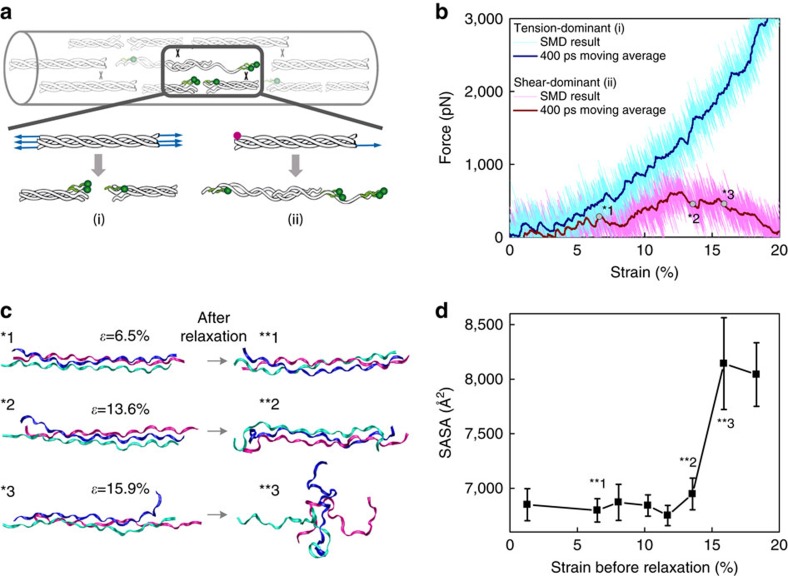
Simulations of tensile strain applied to collagen triple helix. We investigated two possible mechanisms for damage to the collagen triple helix under tension that would allow CHP binding: peptide backbone break of one or more α-chains, referred to as the tension-dominant case, and pull-out of a single α-chain, referred to as the shear-dominant case. These cases were investigated using steered MD simulations for a homotrimeric collagen model peptide made of 10 Gly–X–Y units derived from the crosslink region (930 Lys) of rat and mouse type I, α1 collagen chain. (**a**) Schematic of the two possible loading mechanisms that were investigated in the numerical simulations: the tension-dominant case (i) and the shear-dominant case (ii). (**b**) The force–strain curves of the collagen molecule under tension-dominant and shear-dominant loading. The thicker curves (dark blue and dark red) are results of a moving average with a 400 ps window width. (**c**) Simulation snapshots taken before and after each relaxation simulation for the shear-dominant case. Starting conformations were obtained after a specific level of strain (*ɛ*) in the shear-dominant test as indicated in panel **b**. Water molecules and ions are not shown for clarity. (**d**) SASA, a measure of triple helix unfolding, as a function of applied strain for the shear-dominant case. Structural changes to the triple helix started to take place at 13.6% strain, and by 15.9% strain nearly maximum SASA was reached. Mean±s.d., computed during the last 20 ns of relaxation for each simulation.

We performed computational simulations to gain insights into the potential molecular mechanism of triple helix unfolding under tension that permits CHP binding. Although there are a variety of ways that the collagen molecule could unfold^19^,^43^,^44^,^45^,^46^,^47^, our prior research^19^,^48^ and the organization and crosslinking of collagen at the molecular level in tendons suggest two likely loading mechanisms for irreversible triple helix unfolding that allow for CHP binding (Fig. 6a): tension-dominant loading that stretches the whole triple-helical collagen molecule, as a result of direct stress transfer from uniaxial loading of the fascicle, and shear dominant loading that corresponds to pulling a single strand out of the triple helix, which results from molecular sliding between neighboring collagen molecules, mediated by stress transfer through crosslinks that connect collagen strands of neighboring triple helices. We used massively parallel steered MD simulations to apply either tension-dominant (i) or the shear-dominant loading (ii) to a homotrimeric collagen model peptide with the sequence comprised of ten [Gly–X–Y] triplets derived from the location near the 930 Lys intermolecular covalent crosslink within the triple-helical region of rat and mouse, type I α1 collagen chain^49^. The predicted force–strain curves (Fig. 6b) indicated that the shear-dominant mode leads to irreversible failure at 12.7% strain with a peak force of 621.4 pN, while the tension-dominant mode results in only short-range unwinding of the triple helix as reported by others^43^ and no sign of irreversible global unfolding or failure even up to 20% strain and 3,000 pN force. The shear-dominant mode's peak strength was approximately an order of magnitude lower than the mechanical strength of a covalent crosslink (6–8 nN)^50^, suggesting that such crosslink-mediated stress transfer can cause collagen molecules to fail by strand sliding at strain levels that are compatible with observed strains at failure for tendon fibrils and fascicles.

The molecular level failure caused by shear-dominant loading also led to irreversible unfolding of the collagen model peptide (Fig. 6c). After a strain level of 13.6% followed by full relaxation, the two ends of the model peptide showed clear signs of unfolding, while a larger strain of 15.9% caused the whole molecule to unfold and collapse during the relaxation process. Solvent accessible surface area (SASA), a measure of triple helix unfolding, indicated that an observable structural change to the triple helix started to take place at 13.6% strain, and by 15.9% strain nearly maximum SASA was reached (Fig. 6d). The newly exposed surface area was mainly comprised of unfolded collagen strands, which are the prime targets for CHP hybridization. A similar simulation using a hyper-stable collagen model peptide [(GPO)_6_(GPA)_2_(GPO)_2_] also indicated that triple helix unfolding by strand sliding as a result of shear-dominant loading takes place long before the tension-dominant loading can cause any type of irreversible damage to the collagen model peptide (Supplementary Fig. 8). This suggests that any triple-helical domains in type I collagen, even those that are highly stable, will denature by the sliding mechanism if they experience such shear-dominant stress. Other closely related simulations for the two sequences (pulling one strand while fixing the other two strands, Supplementary Figs 9 and 10) also showed the same trend. From these multiple tests, we concluded that the shear-dominant-mode induced deformation can introduce irreversible molecular damage regardless of the sequence or the loading conditions at the ends of the strands, with failure strains that are reasonable when compared to the failure strains of the RTT fascicles.

### Molecular unfolding of cross-linked full-length collagen

**Figure 7:**
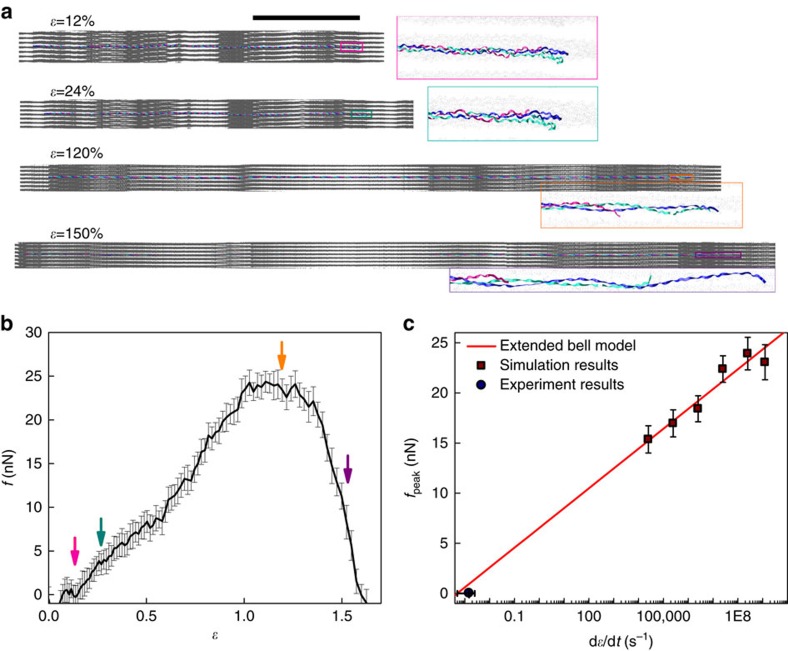
Simulations of tensile strain applied to confined full-length collagen. (**a**) MD simulation snapshots of the mechanical response of the full-length collagen confined in a microfibril under deformations of different strains. Close-ups of the C-terminus of the center molecule are inserted for different strain states with other molecules displayed by gray points as the coordinates of their backbone atoms. Water molecules and ions are not show for clarity. Scale bar, 100 nm. (**b**) The force–strain (*f*-*ɛ*) curve of the center collagen molecule under uniaxial deformation with a strain rate of 10^8^ s^−1^. The plot and error bar are results of average value and s.d. with a 40 ps window width. Snapshots in **a** are indicated by arrows in the plot with corresponding colors. (**c**) The peak force (*f*_peak_) of the *f*–*ɛ* curve measured under different strain rates (d*ɛ*/d*t*) in simulations (mean±s.d.). Data points are fitted according to an Extended Bell Model.

To demonstrate that the molecular mechanism for shear-induced unfolding of a model collagen triple helix is valid for a full-length collagen molecule confined inside a microfibril and interacting with its neighboring molecules, we built a full atomic scale molecular model of a collagen microfibril and repeated the *in silico* mechanical test (Fig. 7a, also see ‘Methods' section and Supplementary Fig. 11). This model considered all interactions with neighboring molecules and loading was applied via the crosslinks and nonbonded interactions with the surrounding collagen molecules, rather than directly to the α-chains of collagen. Results of these simulations demonstrated that the collagen molecule can unfold by mechanical force transmitted through crosslinks once the force reaches a critical peak (Fig. 7b). The critical strain corresponding to the peak force was much larger than reported for collagen microfibrils in experiments for two primary reasons. First, we created only two crosslinks in our model (Supplementary Fig. 11) as this is the smallest number of crosslinks required to generate unfolding of the central collagen; such a limited number of crosslinks contributes to the extensibility of the microfibril structure. Second, the strain rate used in simulations was much larger than the physiological strain rate used in experiments because this made the required computational time for the simulations tractable on currently available high-performance computing platforms. As a consequence, the much higher strain rate significantly increases the predicted peak force and critical strain of collagen. We investigated the strain rate effect on the peak force of unfolding collagen by repeating the tensile tests on the full molecular model and adjusting the strain rate. The peak force was strain rate dependent and followed a logarithmic Extended Bell Model^51^. The extrapolated peak force at a much slower loading rate was in agreement with our experimental measurements (Fig. 7c, filled circle, 18–25 MPa peak force was obtained for 0.0005–0.0025, s^−1^ for collagen with 1.5 nm in diameter). Moreover, at a physiologically relevant loading rate (d*ɛ*/d*t*<1 s^−1^), the peak force, which is the force needed to fail full-length collagen molecule, was much lower than the mechanical strength of a covalent crosslink (6–8 nN). Therefore, the shear-dominant mode of collagen molecular unfolding is a realistic failure mechanism for a full-collagen molecule inside a fibril under tensile loading conditions.

## Discussion

Mechanical damage of collagen fibrils and fibers in connective tissue has been extensively studied by both experiments and computational modeling^11^,^13^,^16^,^17^,^24^,^50^, and as early as 1975, researchers suspected partial denaturation of collagen fibrils during mechanical rupture of human tendons^20^. However, to date, there has been little direct evidence of permanent molecular level damage to collagen, particularly under incremental subfailure conditions, partly because of limitations in collagen imaging methods and unavailability of markers that can recognize unfolded collagen molecules. Using CHP, we directly demonstrated for the first time that irreversible unfolding of collagen molecules is a critical feature of the mechanical failure process in tendon fascicles. In addition, through extensive computational simulations, we identified that shear-dominant stress transfer, which results in pulling one strand out of the collagen triple helix, is a likely mechanism for collagen unfolding during tensile overload, thus allowing CHP binding.

Previous research on damage to collagen across its hierarchy of structure has primarily focused on the level of the collagen fibril^13^,^16^,^32^,^52^. While some evidence existed for damage at the molecular level accompanying damage at the fibril level, this was only suggested for highly damaged tissues and was presented as necessarily preceded by fibrillar disruption^15^,^21^. However, our combined evidence of CHP binding under subfailure loading conditions (Figs 2, 3 and 5), CHP binding to intact fibril structures (Fig. 4b) and simulations demonstrating a likely mechanism for irreversible unfolding of the collagen molecule at strain levels consistent with the experiments, indicate that molecular damage to collagen plays a critical role in tissue damage, occurring at minimum in conjunction with fibrillar disruption and likely preceding it. The CHP binding observed in above-mentioned experiments provides strong evidence against the conventional notion that mechanical overload only disrupts the collagen at its fibril level and that the collagen molecules which are intact but exposed as a result of fibril disruption become partially unfolded and susceptible to trypsin digestion (polymer-in-a box theory)^15^,^21^,^23^. Local unfolding of the so-called ‘thermally labile domain' cannot occur under our experimental conditions because the loading and CHP binding were conducted at 25 °C and 4 °C, respectively, which are far below the 37 °C denaturation temperature of the collagen molecule^22^,^53^. Also, although the imino acid-poor sequences (9–12 amino acids) near the matrix metalloproteinase (MMP) recognition site can adopt a partially unfolded state^44^,^45^, and it was reported that mechanical loading could facilitate the unfolding of such sequences^46^, such regions are too short to form a stable triple helix with CHP, which requires a sequence of at least 6 repeating GPO units^54^. Furthermore, unwinding of these local domains by mechanical stress has been viewed as a reversible process while the molecular damage probed by CHP in this study is apparently irreversible. Our results clearly indicate that collagen molecules themselves are damaged during mechanical loading and that the denatured, non-triple-helical collagen molecules are digested by trypsin in the extraction assay. Furthermore, these results support the most recent work of Veres and coworkers, where differential scanning calorimetry and cold temperature trypsin digestion assay were used to confirm that the collagen molecules in tendon are permanently denatured following tissue mechanical overloading^17^.

By improving the extraction methods for detecting mechanically denatured collagen, we were able to correlate the amount of collagen digested by trypsin with CHP binding even for samples subjected to a single cycle of moderate subfailure loading. To our knowledge, this is the first direct and convincing evidence other than the CHP binding that collagen denaturation is associated with the onset of mechanical damage. Although the improved digestion method yielded quantitative estimation of collagen denaturation, it is a complicated multistep process requiring over 64 h of assay time. CHP-mediated imaging, on the other hand, is a simple and easy method that can reveal location and level of collagen damage from a single fluorescence photograph and the versatility of CHP functionalization allows multi-modality imaging at multiple physical scales, as demonstrated here with fluorescence and electron microscopy. In addition, CHP binding and detection is a non-destructive method with significant potential for diagnostic and therapeutic applications.

Continuous research over more than two decades has focused on explaining molecular level deformation mechanisms of collagen. Based on x-ray diffraction measurements of d-band spacing and orientation, Sasaki and Odajima^14^ proposed a three-stage molecular scale deformation mechanism for collagen, which occurs by molecular stretching, increase in gap distance, and intermolecular sliding. Although this mechanism was mainly used to describe the deformation process in the linear region of the stress–strain curve, and not the failure process during the plateau region of the stress–strain curve, the mechanism is consistent with the results of our simulations, which predict that collagen unfolding during uniaxial tensile loading in tendon is caused by shear-dominant loading at the molecular level. Indeed, our simulations indicate that the small, relatively homogeneous elongation of the collagen triple helix caused by tension-dominant loading is easily recovered during relaxation and does not lead to unfolding of the collagen molecule (Supplementary Fig. 12). Furthermore, breaking covalent chemical bonds from collagen peptide backbone or crosslinks is a highly energetic process that would be expected to result in more catastrophic failure at the tissue level than the gradual accumulation of molecular damage revealed by CHP binding. Previous computational studies suggested that the collagen triple helix could unfold by having individual chains slide past each other under stretching^19^. Based on these prior investigations^55^,^56^,^57^,^58^ and the results of our current simulations showing that the collagen molecule is susceptible to unfolding before cross-link failure in normal physiological loading conditions, it is likely that stretching of a fascicle causes shear-dominant loading of collagen molecules within the fibrils, followed by permanent unfolding of the triple helix via collagen α-chain pullout (Figs 6d and 7a). Here, we demonstrated that the relative shearing of alpha chains with respect to each other, induced by collagen intermolecular cross-linking, can lead to molecular unfolding in the range of strain and loads observed experimentally. Further, simulations using the full-scale collagen model showed that the force transmitted through covalently bonded crosslinks that is necessary to unfold the collagen triple helix is much smaller than the crosslink strength. While the source of application of shear loading can vary (local variation of hydration, salt bridging or other cross-linking mechanisms affecting intermolecular adhesion), together these results demonstrate that shear-dominant loading is one of, if not the most critical mechanism leading to damage to collagen at the molecular level in tendon under uniaxial tension loading. We cannot fully exclude the possibility that the CHP is binding to domains locally unfolded by tension or by fibril disruption; however, the denaturation of the collagen molecule as revealed by CHP cannot be fully explained by any existing mechanisms all of which require the involvement of unstable imino acid-poor domains. This is one of the reasons why we have focused on not local but global unfolding of the collagen molecules as a result of uniaxial stress in our simulations and our simulations have shown that the collagen molecule can be sheared apart under mechanical deformation, suggesting a likely mechanism for triple helix unfolding and hence CHP binding.

Our results provide an explanation for the progression of mechanical damage in collagen-rich tissues, as well as implications for initiation of the inflammatory response and subsequent healing. It is now widely accepted that overuse tendinopathies involve tissue microtrauma or subfailure damage, which causes measurable changes in tissue scale material properties, and often progress to observable and symptomatic damage in the form of partial and full tendon tears^1^,^2^,^3^,^4^. Our cyclic fatigue experiments demonstrated that collagen molecular damage can accumulate with repeated subfailure loading at quasi-static and physiological loading frequencies (Fig. 5) and that this molecular level damage is correlated with changes in tissue material behaviour (Supplementary Fig. 2). These results support previous studies on fatigue damage in tendon that demonstrated low-level fatigue damage, indicated by changes in material behaviour and tissue morphology, after only 160 cycles of creep-fatigue loading that reached peak strains of 6–7% (ref. 32) and reductions in modulus and failure stress for tendon subjected to 300 cycles of creep-fatigue loading to 25 or 60% of the tissue tensile failure stress^52^,^59^. This evidence of low-cycle fatigue damage in tendon, CF-CHP binding accumulation with repeated subfailure loading, and collagen molecular damage in the absence of fiber-scale tissue disruption suggest that subfailure damage may initiate as collagen triple helix unfolding long before progressing to disruption or damage of structures at larger physical scales, and collagen unfolding may even precede detectable changes in tissue material behaviour. Such unfolding of collagen molecules can expose cryptic cell binding domains that are known to elicit healing responses, which include recruitment of cells, removal of damaged collagens and production of new collagen^60^,^61^. A recent study even identified a group of macrophage-like cells that only bind to collagen after denaturation by mechanical overload^61^.

CHP binding provides an exciting and unprecedented tool for detection and quantitative comparison of mechanical damage to collagen in connective tissues, and offers the potential for translational application to diagnosis, staging and treatment of numerous connective tissue disorders and diseases. Standard antibody staining methods that rely on epitopes of a few amino acid residues do not provide sufficient binding sensitivity to distinguish damaged collagen molecules from intact molecules (see Supplementary Methods and Supplementary Fig. 13). Unlike antibodies that are specific to different types of collagens, CHP targets any denatured collagen strands with triple-helical propensity^30^,^62^, which is shared by all types of collagens^63^. Thus, CHP targeting could potentially be used for *in vivo* detection of collagen damage associated with traumatic injuries, for the initial detection and monitoring of diseases such as osteoarthritis and tendinosis, as well as detection of mechanical damage to collagenous tissues caused by extreme mechanical conditions such as tendon fatigue before tissue failure under cyclic loading. Since CHP can be readily conjugated to bioactive molecules^26^,^30^, it could also serve as a targeting moiety to deliver therapeutic agents to sites of injury. These translational applications provide exciting topics for further research and development.

## Methods

### CHP synthesis

All peptides were synthesized by automated solid phase peptide synthesis using TentaGel R RAM resin with 0.18 mmol g^−1^ reactive site density. For CF-CHP synthesis, the sequence GGG(GPO)_9_ was completed by *N,NN′,N′*-tetramethyl-*O*-(1H-benzotriazol-1-yl)uronium hexafluorophosphate (HBTU) chemistry, followed by on-resin labeling with 6 molar equivalents of CF, activated by 6 molar equivalents of PyAOP in NMP for over 24 h. The full-length fluorescent CHP was cleaved from the resin by treating the resin with trifluoroacetic acid (TFA)/triisopropylsilane (TIS)/H_2_O (95:2.5:2.5) for 3 h, and the cleaved peptide was purified by reverse phase HPLC on a semi-preparative Vydac C18 column using a linear gradient mixture of water (0.1% TFA) and acetonitrile (0.1% TFA; 5–45% acetonitrile gradient in 40 min). The purified peptide was subsequently lyophilized, confirmed for purity by matrix assisted laser desorption ionization time of-flight mass spectrometry (UltrafleXtreme, Bruker Daltonics), and reconstituted in 1 × phosphate-buffered saline (PBS).

For synthesizing gold nanoparticle conjugated CHP (NP-CHP), the sequence C(GPO)_9_ and the scrambled sequence (CPGOGPGPOPOGOGOPPGOOPGGOOPPG) were prepared by automated solid phase peptide synthesis, purified by reverse phase HPLC and their molecular weight confirmed using matrix assisted laser desorption ionization time of-flight mass spectrometry, as described above. Purified C(GPO)_9_ was mixed with 10 nm citrate-stabilized gold nanoparticle solution (Ted Pella, Redding, CA, USA), giving a final concentration of 25 μM CHP and 5 nM gold nanoparticles, respectively. The CHP and gold nanoparticle reaction mixture was incubated overnight at room temperature and unreacted C(GPO)_9_ was removed by repeated centrifugation and washing with deionized water.

### Mechanical testing

RTT fascicles were dissected from Sprague Dawley rat tails (rat tails purchased from BioreclamationIVT, Westbury, NY, USA) by holding the proximal tail end while gripping the distal end with a hemostat. The hemostats were twisted around the axis of the tail until the skin separated, at which point the distal end was pulled away from the tail revealing tendon fascicles. The fascicles were cut away and individual fascicles were tested by one of the two protocols in a mechanical tester. All testing was performed on a custom micro-materials test system (Supplementary Fig. 1a) consisting of a brushless servomotor, lead-screw driven linear stage controlled by a custom LabVIEW Virtual Instrument graphical interface, and an adjustable fixed support to ensure proper sample alignment. Samples were gripped using custom machined aluminum clamps containing a grooved 3D printed polymer clamping surface (Supplementary Fig. 1b) for secure gripping of samples without causing failure at the clamping surface. A sample bath was attached to the test rig to ensure sample hydration throughout mechanical testing.

Tensile tests were performed by loading individual fascicles into the mechanical tester sample clamps and applying a 0.03 N preload to remove slack from the samples and provide a consistent point for the zero-strain length measurement^9^,^32^. Fascicles were then strained at 0.5% per second to 1.0, 2.5, 5.0, 7.5, 9.0, 10.5, 12, 13.5 or 15% strain, as measured from displacement of the clamps (*n*=3 samples for each strain group). Unloaded fascicles were used as a negative control (*n*=5). All tests were performed at room temperature with samples immersed in 1 × PBS. For stress calculations, the fascicles were assumed to have a circular cross-section and the average diameter of each fascicle was calculated from 5 measurements along the length of each fascicle.

Cyclic tests were performed by loading individual fascicles into the mechanical tester sample clamps followed by application of a 0.03 N preload. Fascicles were preconditioned for 10 cycles at 1 Hz from 0 to 4% strain, and 0.03 N preload was reapplied to give a new zero-strain length. Immediately following preconditioning, fascicles were loaded at 1 Hz from 1 to 5% strain for 10, 100 or 1,000 cycles or at 0.1 Hz from 1 to 5% strain for 10 or 100 cycles (*n*=3 samples for each group). The control groups for the fatigue testing consisted of unloaded (*n*=3) and preconditioned-only (*n*=3) fascicles. All tests were performed at room temperature with samples immersed in 1 × PBS.

### CF-CHP staining of tendon fascicles

Loaded and unloaded-control fascicles were stained with CF-CHP to detect the presence of collagen damage. Because the CHP slowly self-assembles into its own triple helix over time and loses the driving force for collagen hybridization, the trimeric CHP must be thermally dissociated to monomers before use^25^. To prevent artificial thermal damage to the fascicles, the hot CHP solution was quickly cooled down to room temperature and diluted to a low concentration followed by immediate application to the tissue (dead time <1 min). In this way, most CHP peptides are expected to remain as active monomers during the staining process, based on previous kinetic studies on CHP triple helix folding^64^ and kinetic calculations^65^. CHP trimerization follows a third order reaction rate that is drastically slow at low concentrations^66^. CF-CHP stock solution (150 μM) was heated at 80 °C for 10 min to thermally dissociate trimeric CHP to a monomeric state, and quenched by immersion in 4 °C water for 15 s. Each fascicle was placed in a vial containing 475 μl of 1 × PBS, and 25 μl of monomeric CF-CHP stock was added, resulting in a final CF-CHP concentration of 7.5 μM. Fascicles were incubated overnight at 4 °C and then unbound CF-CHP was removed by washing 3 times in 1 ml of 1 × PBS for 30 min at room temperature. Fascicles were stored in frozen 1 × PBS at −20 °C until imaging, which was demonstrated to not affect the CF-CHP fluorescence intensity (Supplementary Fig. 14).

### Fluorescence imaging and analysis

Stained fascicles were imaged for CF fluorescence intensity using an automated Nikon Ti-E inverted wide-field fluorescence microscope. CF-CHP stained fascicles were unloaded from the micro-materials test system, mounted in 1 × PBS between a glass slide and a glass cover slip, and imaged with a 4 × objective lens using the fluorescein isothiocyanate (FITC) channel and 488 nm excitation. Images of whole samples were captured by CCD camera at 16-bit depth using the built-in large image acquisition feature.

Wide-field fluorescence images were quantified for fluorescence intensity using a thresholding segmentation method. Micrographs of tensile test samples were thresholded by Otsu's method^67^ to identify fascicle regions with CF-CHP staining. Fatigue test sample micrographs were thresholded by subtracting the average pixel value of the unloaded, CF-CHP stained control samples, and the mean value of the remaining positive valued pixels was calculated. The mean pixel intensity value was used as the measurement of CF-CHP staining intensity. The two different segmentation methods were used based on the different CF-CHP staining patterns exhibited by the tensile loaded and cyclically loaded samples. Specifically, the automated thresholding method isolated the stained areas at the grip interface, making it unable to capture changes in staining across the entire tested region of the sample, requiring the use of background subtraction. All statistical analyses were performed using SigmaPlot 11.0 (Systat Software, Inc., San Jose, CA, USA). All sample groups were compared to experimental controls using *t*-tests with the Holm–Sidak correction for multiple comparisons, with a significance of *α*=0.05.

### Second harmonic generation and multiphoton microscopy

Samples that were subjected to tensile testing were imaged by two-photon confocal microscopy, using a custom Prairie View Ultima multiphoton microscope (Bruker Corp., Billerica, MA, USA) to localize CF-CHP binding within the tissue. Fascicles were mounted in Fluoroshield mounting media (Sigma-Aldrich, St Louis, MO, USA) and imaged with 910 nm excitation and detection in 435–485 nm and 500–550 nm for collagen SHG and CF two-photon excited fluorescence, respectively.

### NP-CHP assisted TEM

Samples were stained with NP-CHP for TEM study to understand the location and condition of CHP binding on collagen fibrils. Tissue sections of damaged and undamaged RTT fascicle were prepared as described by Starborg and coworkers^68^. Briefly, one 12% strain fascicle and one intact unstrained fascicle were fixed overnight in PBS buffer solution containing 2.5% glutaraldehyde and 1% paraformaldehyde at 4 °C. From each fascicle, three small pieces were dissected from three different locations along the long axis of the tissue. The dissected pieces were first stained by tannic acid, then osmium, and finally by uranyl acetate. After each staining step, the samples were washed with deionized water five times. The samples were then dehydrated using 30, 50, 70, 90 and 100% graded ethanol for 10 min at each step, followed by embedding in 100% Agar100 Hard in labeled molds. Samples were allowed to cure at 45 °C for 72 h. Sections of 70–80 nm thickness were cut and mounted onto carbon-coated TEM grips. Each TEM grip contained at least five sections. Each section was stained by incubating in a solution of 10 nM NP-CHP in 2% bovine serum albumin for 15 min at room temperature. Following incubation, sections were washed 6 times with room temperature deionized water. NP-CHP labeled tissue sections were imaged by TEM using a FEI Tecnai T12 microscope (FEI, Hillsboro, OR, USA) operated at 120 kV.

Three groups of samples were stained using NP-CHP: unstretched RTT fascicle, RTT fascicle stretched to 12% strain, and RTT fascicle stretched to 12% strain and stained with a scrambled sequence NP-^S^CHP (*n*=5 images per group). Staining density of NP-CHP was quantified by the areal density of gold nanoparticles on TEM images, defined as the number of nanoparticles per unit area (# μm^−2^). For all images, areal density of NP-CHP was determined as the total number of nanoparticles in the image divided by the total image area. Additionally, 12% strain images stained with NP-CHP were quantified to compare areal staining density on undamaged (regions retaining characteristic d-banding pattern) and damaged (regions missing d-banding pattern) areas. All analysis of TEM images was performed in ImageJ; nanoparticles were highlighted and counted using the ‘mark-points' tool and the size of undamaged tissue in the 12% strain images was measured by creating a selection that included all tissue with the characteristic d-banding pattern. T-tests were performed to compare unloaded and loaded tissues, damaged and undamaged regions of loaded tissue, and unloaded tissue stained with NP-CHP and loaded tissue stained with scrambled sequence NP-^S^CHP. The Holm–Sidak method was used to correct for multiple comparisons.

### Simulation of collagen model peptides under tensile loading

The computational model of the collagen triple helix molecule represented type I collagen from *Mus musculus* with the sequence encoded as GEV GPO GPO GPA GEK GSO GAD GPA GSO GTO, which is close to the location of the known intermolecular covalent crosslink at position 930 (ref. 49). The N-terminus was acetylated and C-terminus was capped with N-methylamide to remove charges. The molecule was equilibrated in a water solution of 0.1 mol l^−1^ NaCl and the ratio between cations and anions was chosen for neutral system charge. Periodic boundary conditions were applied to all directions, and the system size was maintained at 18 × 5 × 5 nm to keep the minimum distance between mirroring images larger than the cutoff distance. Each simulation was performed in the isothermal–isobaric ensemble (normal temperature and pressure (NPT), constant pressure of 1 atm and constant temperature of 310 K) by using LAMMPS open-source code with interatomic interactions defined by the CHARMM22 force field for proteins and solvent. The simulation time step was 2 fs using a rigid bonds model for all of the covalent bonds between hydrogen atoms and other heavy atoms. Energy minimization and equilibrium simulation was performed for 22 ns for a fully equilibrated structure before mechanical test. A particle mesh Ewald function was used with a grid width <1 Å to calculate the electronic interactions. The steered MD method was used to deform the molecule with a constant loading speed at 0.002 Å ps^−1^. The triple helix collagen molecule was deformed by fixing one alpha carbon atom at the N-terminus of one chain and tensile force is applied to an alpha carbon atom at the C-terminus of another chain. After a certain amount of tensile strain, the structure was taken out and allowed to stress relax for 40 ns. SASA was computed during the last 20 ns of the relaxation simulation using the VMD Molecular Graphics Viewer^69^ with a probe radius of 1.4 Å. We examined additional boundary conditions for the N and C-terminus ends of the triple helix as well as a more thermally stable molecular sequence [(GPO)_6_(GPA)_2_(GPO)_2_], and the results of these simulations are detailed in Supplementary Figs 8–10 and Supplementary Table 1.

### Tensile loading simulation of full-length collagen molecule

Four collagen molecules were explicitly included in the system and full periodic boundary conditions were applied in all directions. Each collagen molecule with ∼300 nm in length was built according to the coordinates of alpha carbon atoms based on Protein Data Bank entry 3HR2. Two crosslinks, K87 and K930, were created as shown in Supplementary Fig. 11 for the central collagen molecules, as they represent two known intermolecular divalent enzymatic crosslinks at two ends of a collagen molecule^49^. The initial size of the unit cell was 9 × 9 × 322 nm with a tilt angle of 30° for its cross-section geometry. A water solution of 0.1 mol l^−1^ NaCl was used to fill the gap between collagen molecules, and the system was equilibrated in a NPT ensemble for 6 ns before subjecting to tensile tests. Deformation was applied to the entire system with a constant strain rate during a dynamic run while the system could simultaneously relax. The movement of the alpha carbon atom at the far end of each crosslink was remapped by the deformation of the system for each time step to ensure that the central collagen was deformed with a constant strain rate under a full control. The forces in two crosslinks were averaged and recorded during the loading test.

### Data availability

The data that support the findings of this study are available from the authors upon reasonable request. The LAMMPS open-source code is publicly available at http://lammps.sandia.gov.

## Additional information

**How to cite this article:** Zitnay, J. L. *et al*. Molecular level detection and localization of mechanical damage in collagen enabled by collagen hybridizing peptides. *Nat. Commun.* **8,** 14913 doi: 10.1038/ncomms14913 (2017).

**Publisher's note:** Springer Nature remains neutral with regard to jurisdictional claims in published maps and institutional affiliations.

## Supplementary Material

Supplementary Information Supplementary Figures, Supplementary Table, Supplementary Methods and Supplementary References
