# Supplementary material for: Molecular level detection and localization of mechanical damage in collagen enabled by collagen hybridizing peptides
Source: Nat Commun. 2017 Mar 22;8:14913. doi: 10.1038/ncomms14913 (PMC5364439; doi:10.1038/ncomms14913)
Supplement: Supplementary Information — Supplementary Figures, Supplementary Table, Supplementary Methods and Supplementary References [file ncomms14913-s1.pdf]

SUPPLEMENTARY FIGURES

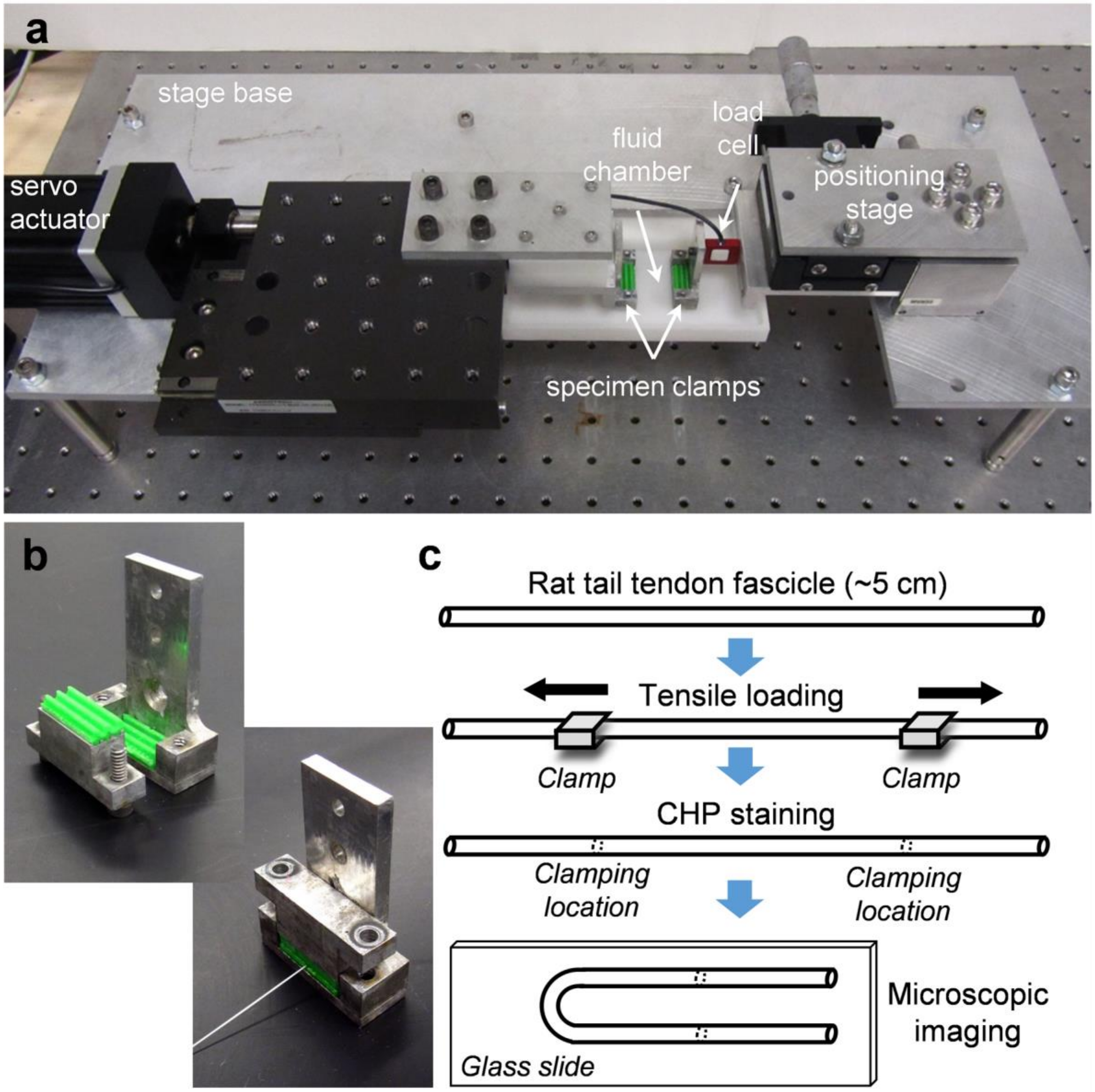

Supplementary Figure 1. Mechanical test system and testing schematic. (a) A photograph of the micro-mechanical system for creation of damage and failure in rat tail tendon fascicles. The system uses an Aerotech ATS03005 precision ground lead screw and brushless servomotor stage (Aerotech, Inc., Pittsburgh, PA, USA) to displace specimen clamps. Custom-machined aluminum specimen clamps, with 3D printed plastic jaws having interlocking square teeth, were used to grip fascicle samples (close up photographs shown in b). Sample baths were machined out of Delrin acetal resin. Force was measured using a calibrated FUTEK LSB210, 2 lb submersible s-beam load cell (FUTEK Advanced Sensor Technology, Inc, Irvine, CA, USA). The test system was attached to a machined aluminum plate and mounted on an optical breadboard air-table for vibration isolation. (c) Schematic of the experiment workflow. The approximate clamping locations on a fascicle and the way the fascicle is folded and positioned on a glass slide during microscopic imaging are shown. Fluorescence and brightfield images of the stained fascicles are shown in Figure 2b.

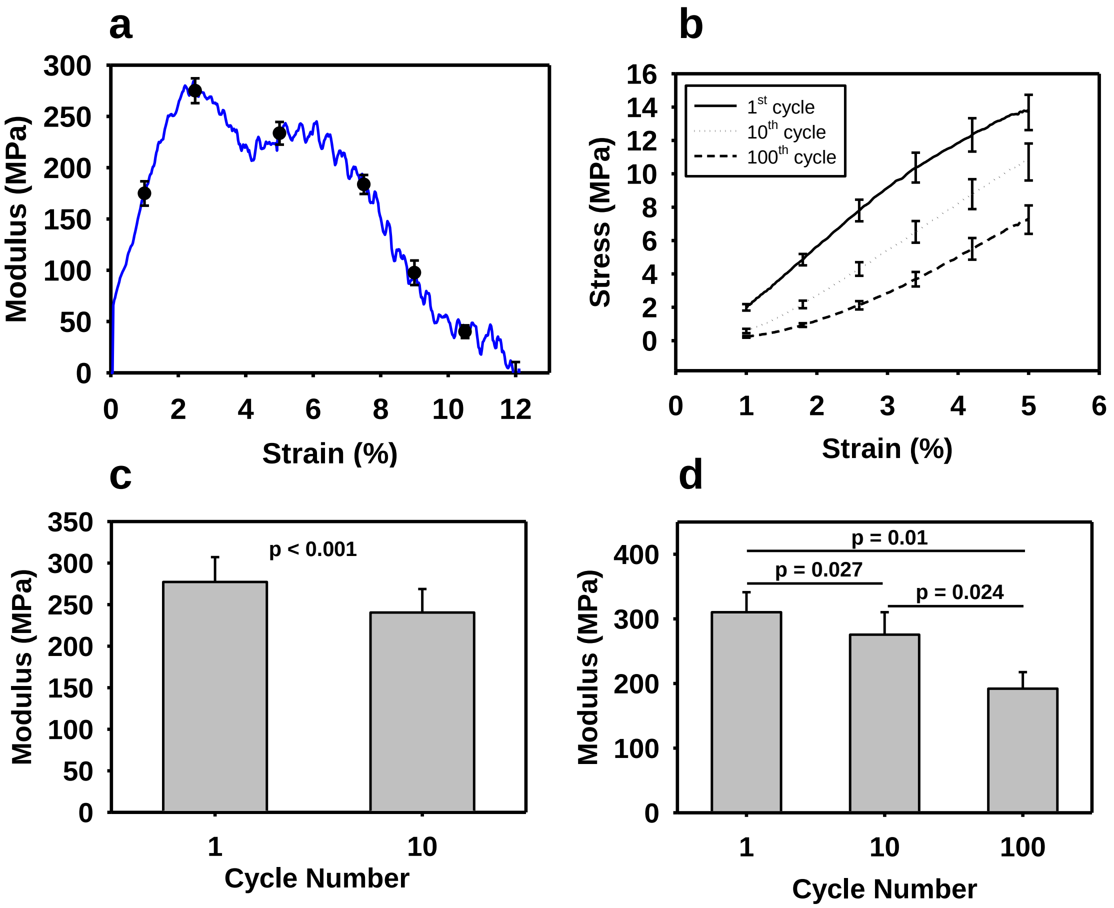

<u>Supplementary Figure 2.</u> Changes in material properties as a function of strain level and cycle number. (a) Incremental modulus as a function of applied strain for the monotonic testing across all samples that reached the particular strain level. E.g., from 0-1% strain there were 27 samples, at strains between 5-7.5% there were 18 samples, etc. The modulus at each strain level was calculated using the data points at ±0.075% strain. Individual data points with standard errors represent the specific strain levels analyzed for CHP binding. The maximum modulus occurred at 2.5% strain, and all other moduli were significantly lower than this maximum ($p \leq 0.01$). Mean ± s.e.m. (b) Stress-strain curves from the loading part of the cycle for the cyclic fatigue samples tested at 0.1 Hz to 100 cycles (n = 3, mean ± s.e.m.). (c) Comparison of modulus calculated from the loading part of the stress-strain curve for the cyclic fatigues samples tested at 0.1 Hz to 10 cycles (n = 6, mean ± s.e.m.). Ten cycles of loading produced a significant decrease in modulus. (d) Comparison of modulus calculated from the loading part of the stress-strain curve for the cyclic fatigues samples tested at 0.1 Hz to 100 cycles, corresponding to the stress-strain curves in panel b (n = 3, mean ± s.e.m.). There was a significant decrease in modulus at 10 and 100 cycles in comparison to the first cycle, and there was a significant decrease in modulus between 10 and 100 cycles.

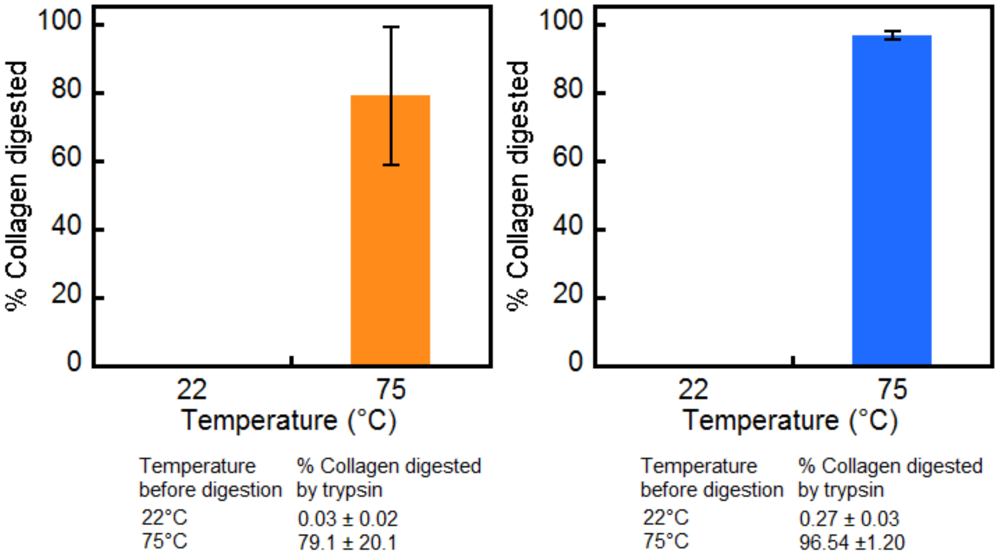

<u>Supplementary Figure 3.</u> Results of trypsin digestion assay obtained using the literature method (left) and the improved method (right). Unstretched rat tail tendon fascicles were incubated at 22 °C (intact) or 75 °C (heat denatured) for 10 min prior to the assay (n = 5 each group). The percentage of the trypsin-digested collagen in each fascicle was calculated from quantification of the collagen contents of the trypsin solution and the undigested remaining part using the hydroxyproline assay (mean ± s.d.). The improved method gave an order of magnitude lower statistical error compared to the literature method.

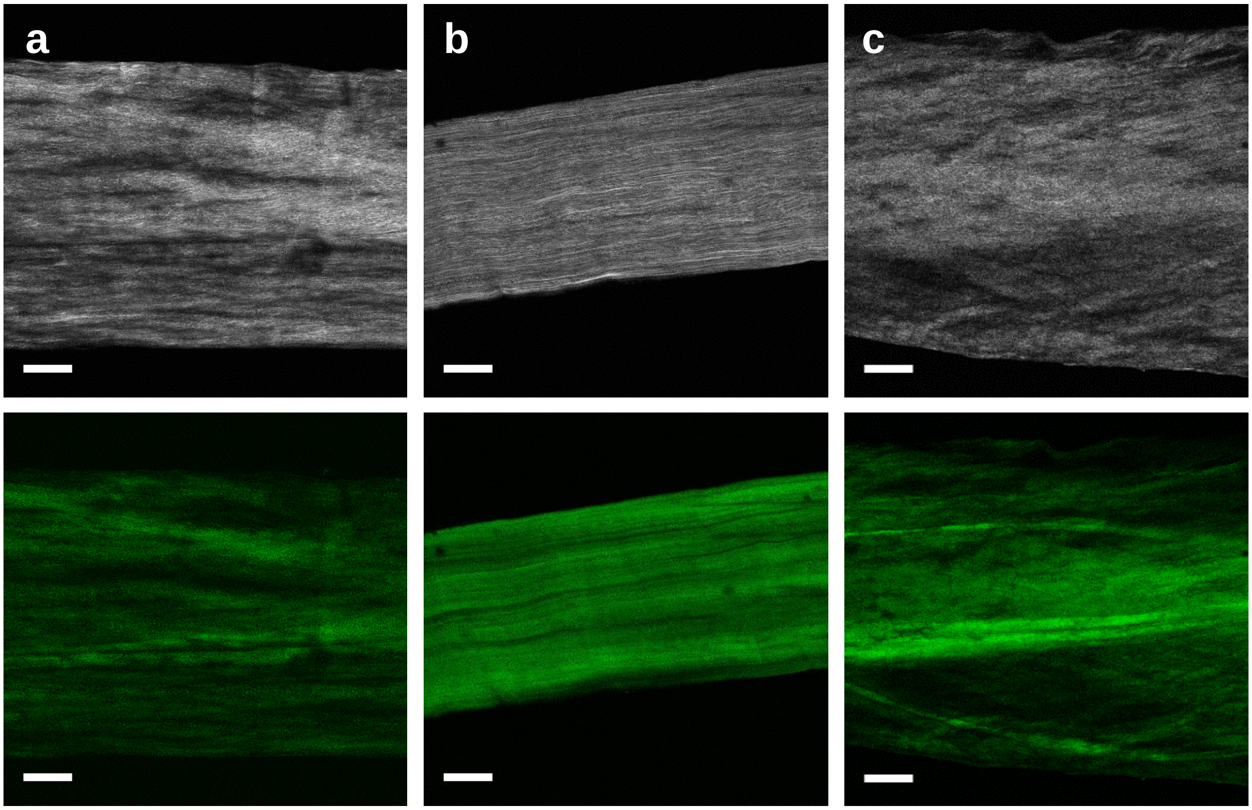

<u>Supplementary Figure 4.</u> CF-CHP staining produces heterogeneous banded patterns at multiple strain levels. In multiphoton images of rat tail tendon fascicles (SHG signal shown in white, top), the banded staining pattern of CF-CHP (TPEF signal shown in green, bottom) was observed in samples with different strain levels (a-c), indicating that there was heterogeneity in damage distribution at all strain levels. (a) 7.5% strain, (b) 13.5% strain, (c) 15% strain. Scale bars: 100 μm.

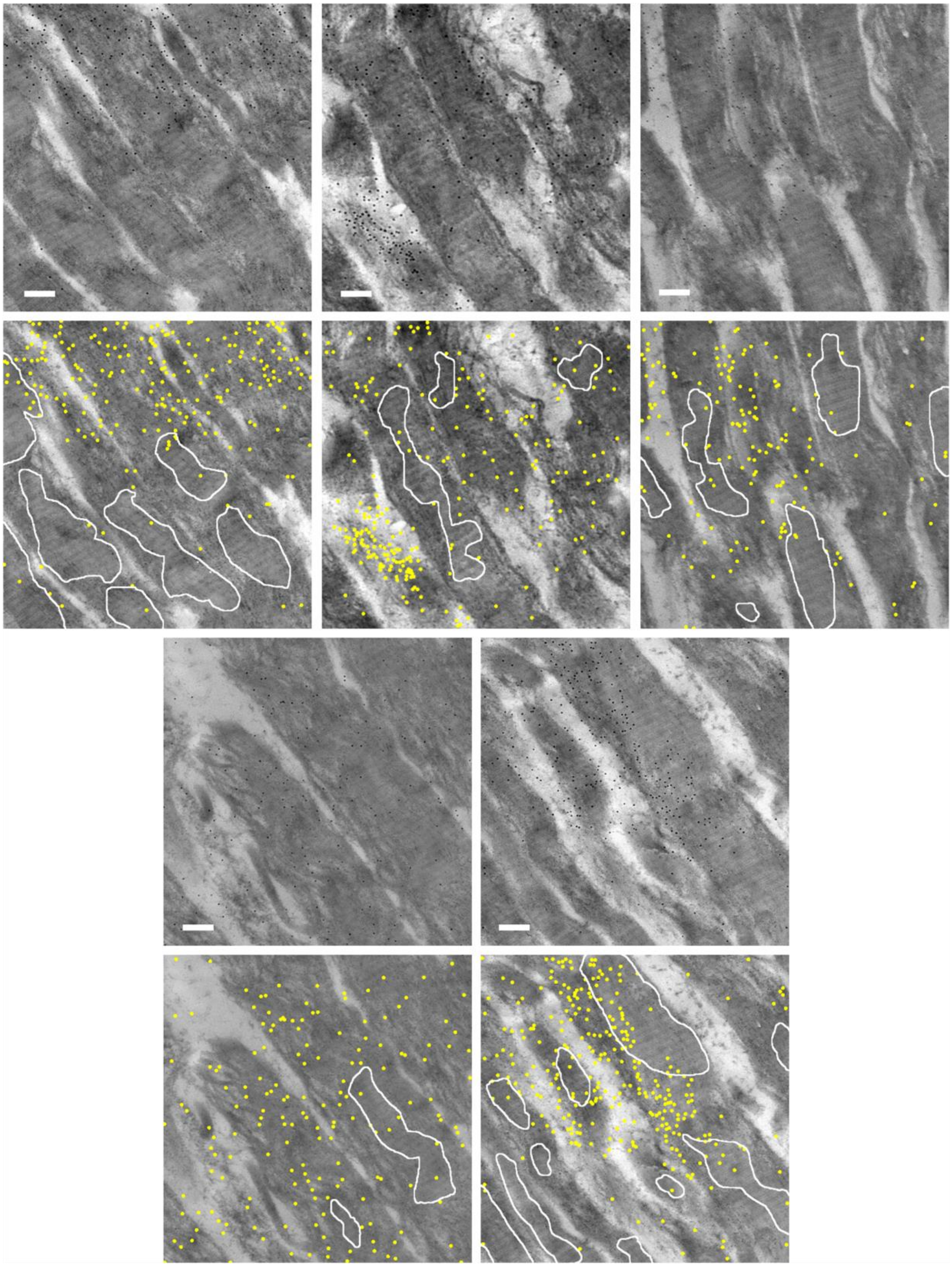

<u>Supplementary Figure 5.</u> Transmission electron micrographs of NP-CHP binding to damaged collagen fibrils in a 12% strain fascicle. Five randomly selected microscopic fields are shown. Dark NP-CHP particles are marked by yellow dots and areas with remaining d-banding pattern are outlined by white lines for clarity in the bottom images. Scale bar: 200 nm.

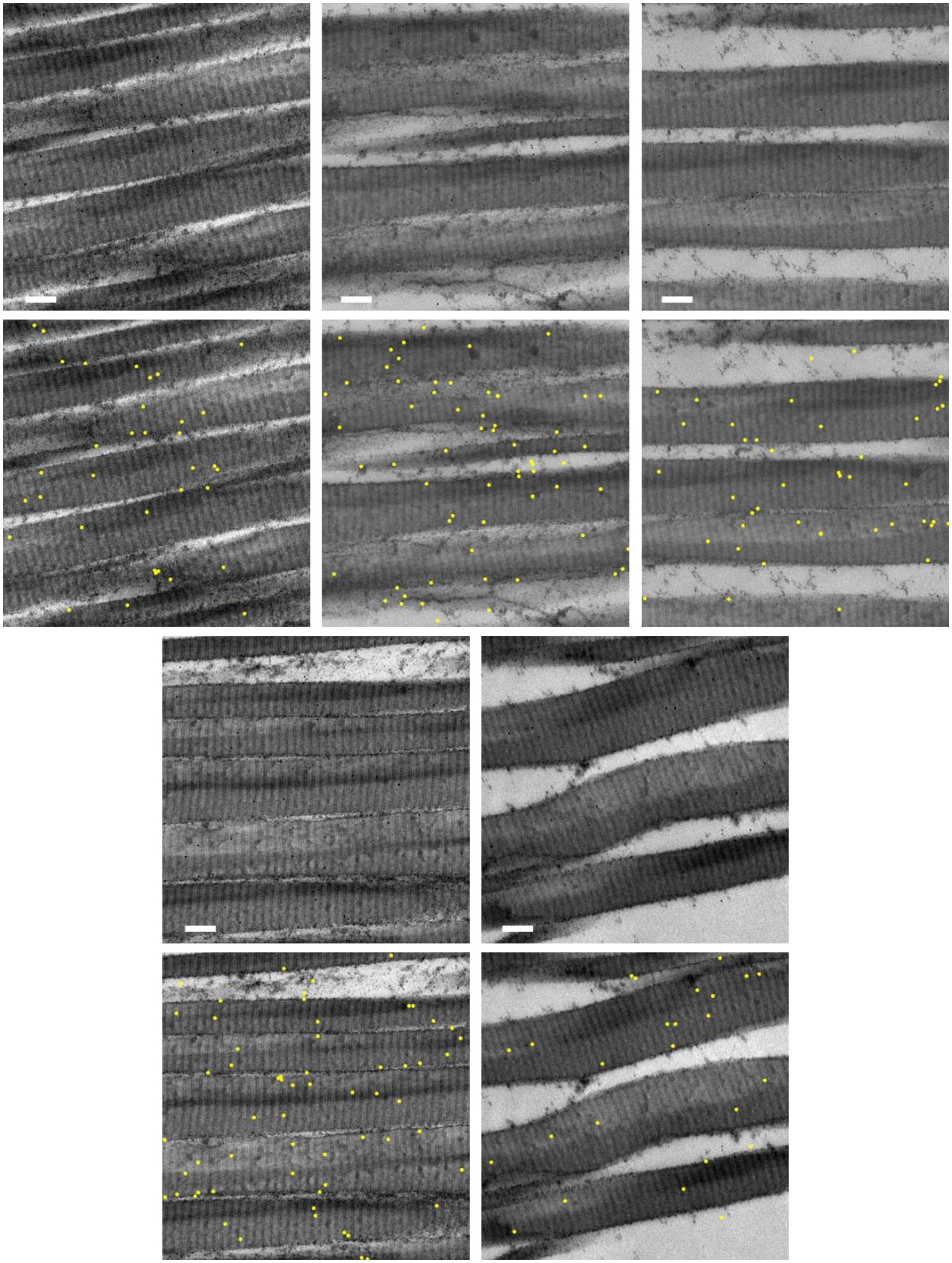

Supplementary Figure 6. **Transmission electron micrographs of NP-CHP stained collagen fibrils in an unloaded fascicle.** Five randomly selected microscopic fields are shown. Dark NP-CHP particles are marked by yellow dots for clarity in the bottom images. Scale bar: 200 nm.

5

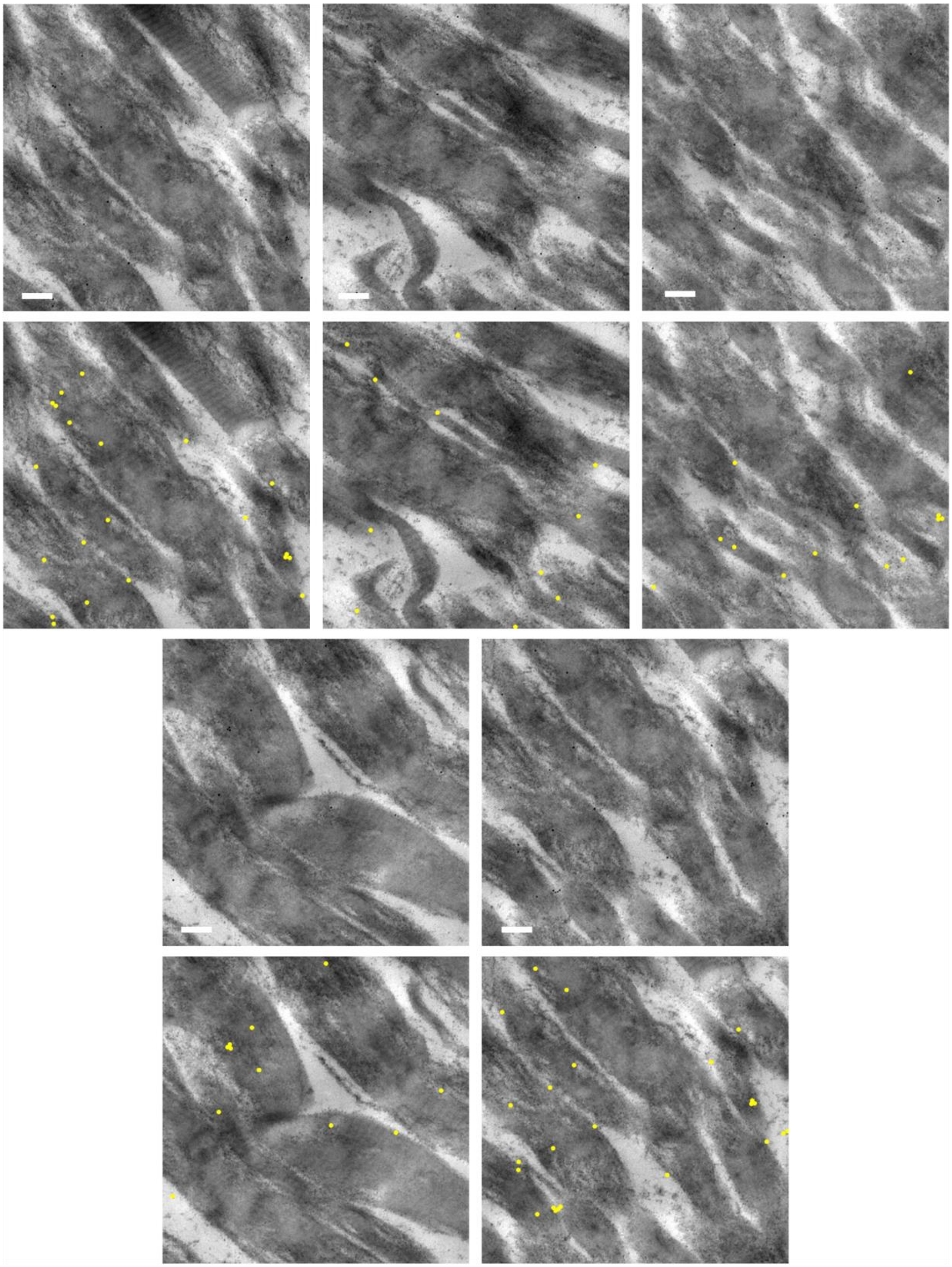

<u>Supplementary Figure 7.</u> Transmission electron micrographs of damaged collagen fibrils in a 12% strain fascicle incubated with scrambled sequence NP-CHP. Five randomly selected microscopic fields are shown. Dark NP-CHP particles are marked by yellow dots for clarity in the bottom images. Scale bar: 200 nm.

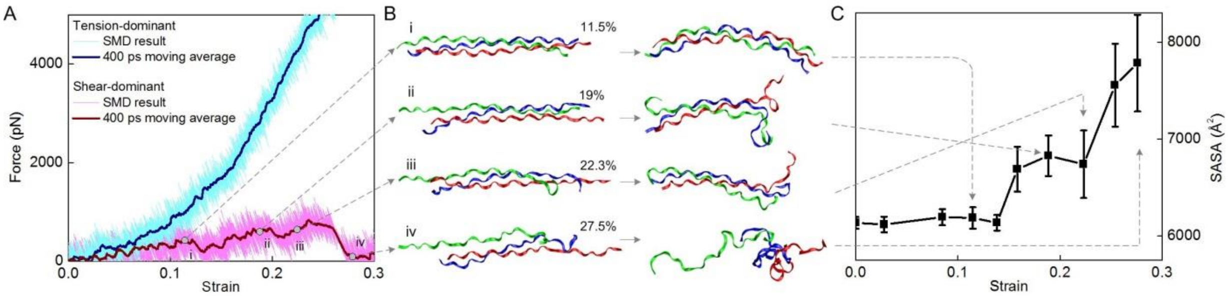

<u>**Supplementary Figure 8.**</u> **Simulation of hyperstable triple helix collagen model peptide.** We examined another collagen sequence [$(GPO)_6(GPA)_2(GPO)_2$] that has high thermal stability which would be more difficult to denature by mechanical forces. The boundary conditions for these simulations were identical to those used for the results in Figure 6. (A) The force-extension curve of the collagen molecule under two different tensile tests as N-terminus of all the 3 chains fixed and C-terminus of all the 3 chains loaded (tension-dominant, top blue curve) and N-terminus of one chain fixed and C-terminus of another chain loaded (shear-dominant, bottom red curve) in SMD simulations. The thicker curves are the results of moving averaging with window width of 400 ps. Comparing results to Figure 6, the strain levels that produced permanent uncoiling of the triple helix increased from 12.7% to 23.5%. (B) Simulation snapshots taken before and after each relaxation simulation. (C) The unfolding process of the collagen molecule corresponds to increment of SASA in a way similar to the data in Figure 6d. The results of these simulations are in general agreement with the findings in Figure 6; shear-dominant loading leads to irreversible failure long before tension-dominant force does, except that levels of peak force and strain are higher because of hyperstability of the triple helix. Mean ± s.d., computed during the last 20 ns of relaxation for each simulation.

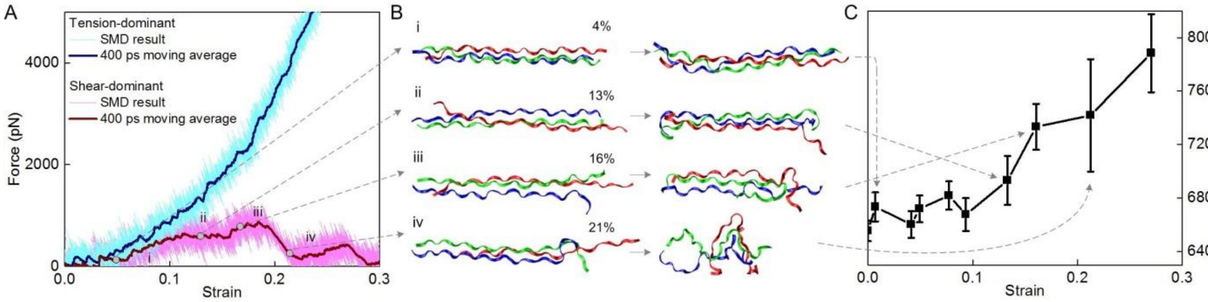

<u>Supplementary Figure 9.</u> Simulation of triple helix collagen model peptide under tension with two chains fixed boundary condition. We simulated the same collagen model peptide representing the crosslinked region of the type I collagen as described in the main text under the additional shear-dominant test condition of pulling on one peptide strand of the triple helix while fixing the other two (rather than fixing one strand as in Figure 6 of the manuscript). (A) The force-strain curves of the collagen molecule under two different tensile test conditions: N-terminus end of all 3 chains fixed and C-terminus of all the 3 chains loaded (tension-dominant, top blue curve same as the blue curve in Figure 6b), and N-terminus of 2 chains fixed and C-terminus of the other chains loaded (shear-dominant, bottom red curve) in SMD simulations. The thicker curves are the results of moving averaging with window width of 400 ps. Peak force was 874.7 pN with the strain of 18.5%. (B) Simulation snapshots taken before and after each relaxation simulation. Starting conformations are obtained after certain amount of deformation in the shear-dominant test as indicated in panel A. Water molecules are not shown here for clarity. (C) Solvent accessible surface area (SASA), a measure of triple helix unfolding, as a function of applied strain for the shear-dominant case. Mean ± std, was computed during the last 20 ns of relaxation for each simulation. The results of these simulations are in general agreement with the findings in Figure 6, but with different levels of force and strain for the shear-dominant case. Mean ± s.d., computed during the last 20 ns of relaxation for each simulation.

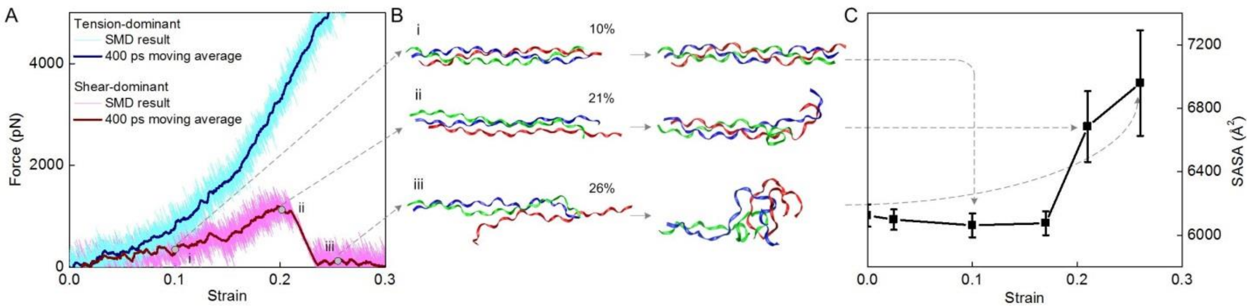

<u>Supplementary Figure 10.</u> Simulation of hyperstable triple helix collagen model peptide with two chain fixed boundary condition. For comparison, we simulated the hyperstable collagen model peptide (same peptide tested in the Supplementary Figure 8) using the same boundary condition as the Supplementary Figure 9 with two N-terminus chains fixed and the C-terminus of the other chain loaded. (A) As can be seen from the bottom red curve, the peak force for this specific sequence and loading condition was 1201.2 pN, which is the highest value for all the shear dominant tests performed. The failure strain corresponding to this peak force was 19.7%, which is also the highest. (B) The triple helix structure of this collagen molecule starts to unfold at strain around 21%, where the chain starts to slide apart from the other two after relaxation. (C) The unfolding process under this loading condition corresponded to a more severe increment of SASA, but followed a similar trend as other sequences. Mean ± s.d., computed during the last 20 ns of relaxation for each simulation.

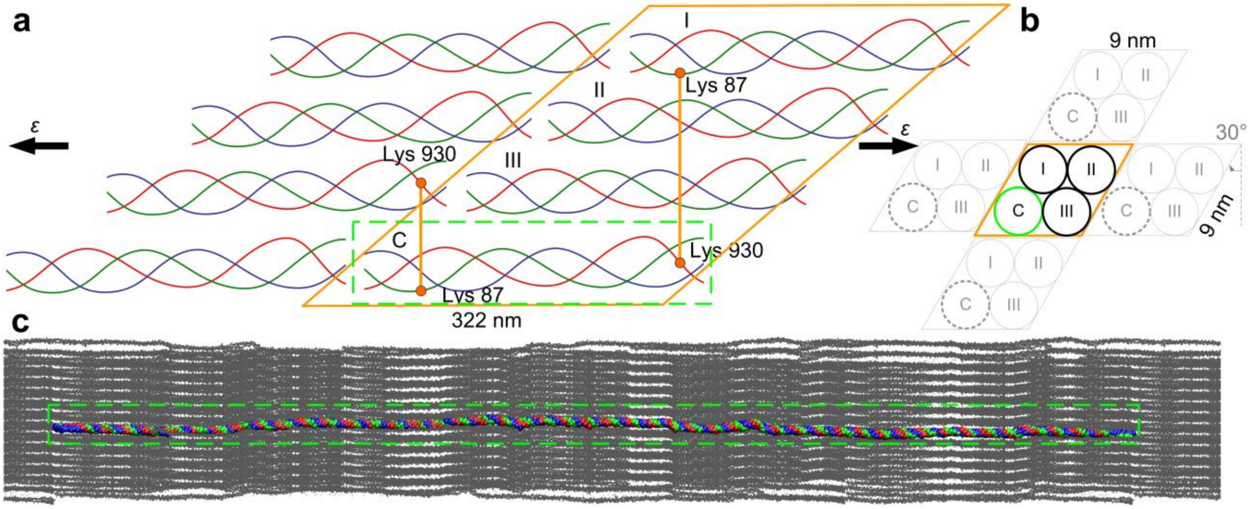

<u>Supplementary Figure 11.</u> Computational model of a full length collagen molecule within a microfibril, and schematics of boundary conditions and crosslinks. (a) Four collagen molecules (I, II, III, C) were explicitly included for the system (highlighted by the solid orange region) and full periodic boundary conditions were applied to all directions. Two crosslinks, one between Lys87 in chain 1 of the center molecule (C) and Lys930 of the molecule III, and the other crosslink between Lys930 in chain 2 of the molecule C and Lys87 of the molecule I, were created for the center collagen molecule C as they represent the two known intermolecular covalent crosslinks around two ends of the collagen molecule. The system size in the collagen length direction was 322 nm. (b) The initial geometry of the cross-section of the unit cell of our model was of a size 9 nm × 9 nm with a tilt angle of 30°. It was shown that with the given periodic boundary conditions, the center collagen molecule was fully covered and interacting with other collagen molecules. (c) A simulation snapshot of the collagen computational model with mirroring simulation systems shown in gray. Molecule C in all the panels is highlighted by green.

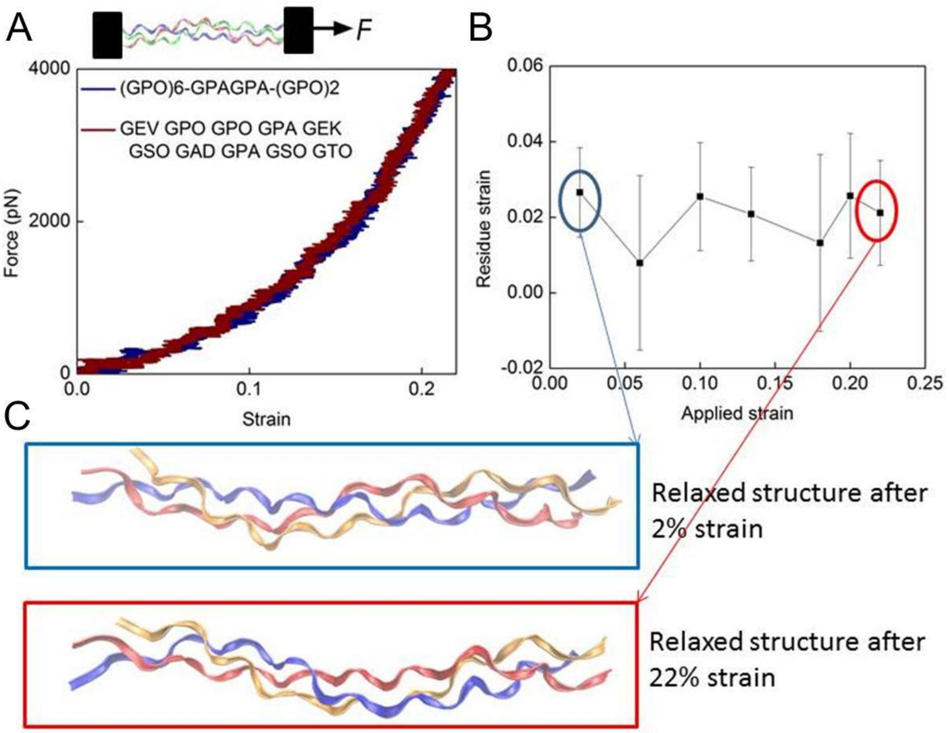

<u>Supplementary Figure 12.</u> Simulation of triple helix collagen model peptide under homogeneous elongation caused by tension-dominant loading. (A) Schematic of the loading boundary conditions, with all the three chains fixed at one end and all three chains loaded at the other end, and the force-strain curves for two collagen molecules with different amino-acid sequences. (B) The collagen model was allowed to relax after elongation by instantaneously releasing the force boundary conditions. The relaxations are initiated from different collagen molecular conformations with various applied strains. Each of the molecular conformations was allowed to fully relax to reach the equilibrated structure as assessed by the convergence of conformation energy. The strain remaining in the molecule was measured for the relaxed molecular structure (mean ± s.d.). (C) There were no significant structural changes after deformation by a large applied strain (22%) from both molecular structure with the given snapshots (solvent molecules and ions are not shown for clarity) and residue strain measurements, suggesting the tension-dominant loading is not sufficient to cause molecular unfolding.

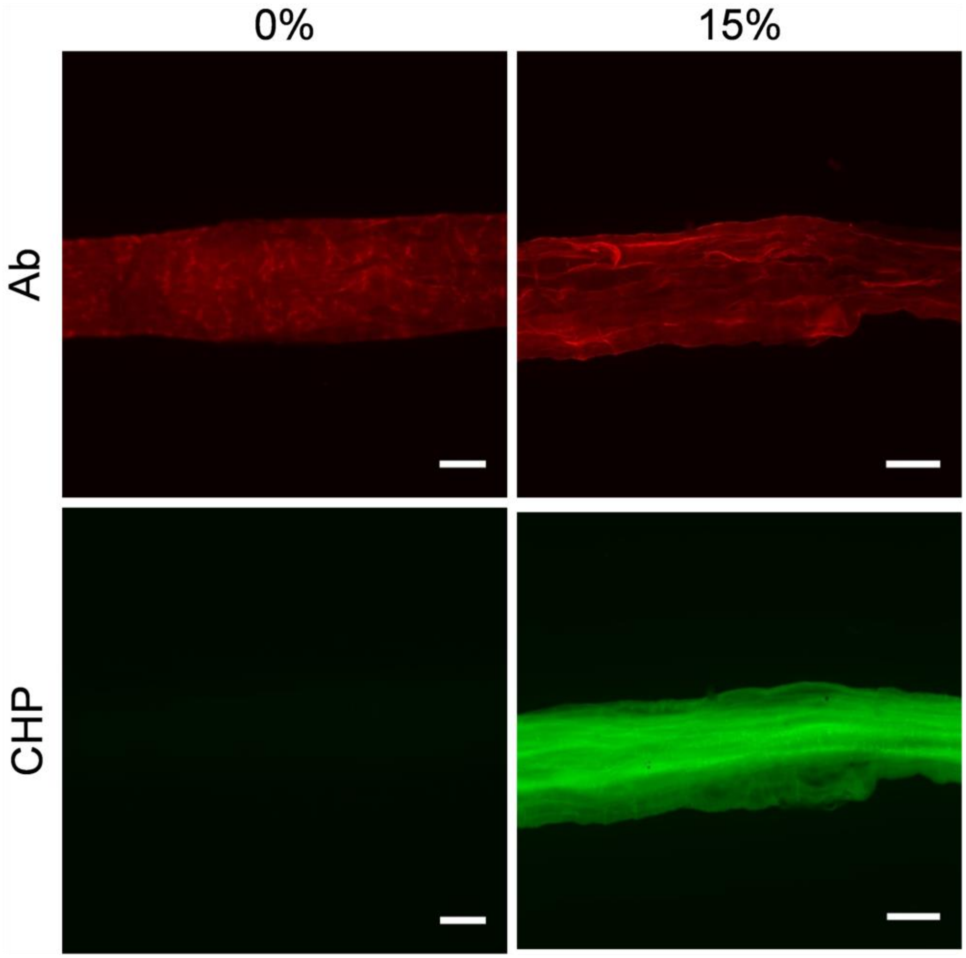

<u>Supplementary Figure 13.</u> Comparison of collagen antibody staining with CHP staining for mechanically stretched fascicles. Images of a pair of unstrained (0%) and highly strained (15%) rat tail tendon fascicles stained with both anti-collagen type-I antibody (top panels, signals shown in red) and CF-CHP (bottom panels, signals shown in green). While the CHP staining exhibits obvious sensitivity to mechanical loading, there are no apparent fluorescence intensity differences between the 0 and 15% strain samples from the antibody staining of the same samples, indicating that the antibody is incapable of distinguishing mechanically damaged collagen molecules from intact molecules. Scale bars: 300 µm.

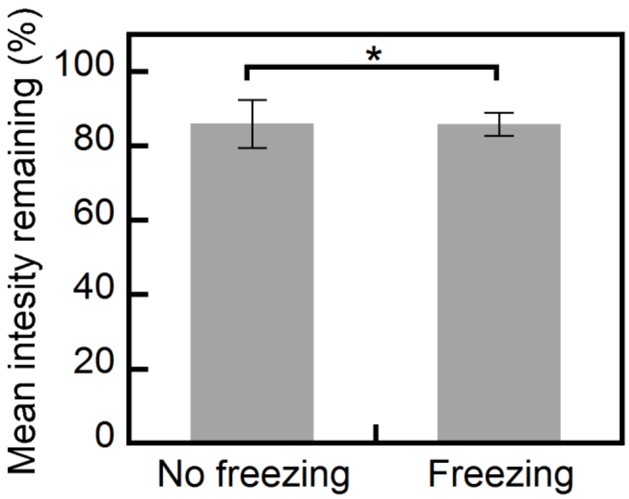

<u>Supplementary Figure 14.</u> The effect of frozen storage on CF-CHP fluorescence intensity is negligible. A group of CF-CHP stained 12% strain fascicles were imaged twice, before and after storage at -20 °C overnight (n = 4, see supplimentary method), and the mean fluorescence intensity quantified from the two sets of images were compared (mean ± s.e.m.). Considering the inevitable photobleaching, another group of fascicles (n = 4) prepared in the same fashion were imaged twice without frozen strorage. Both groups showed a significant reduction (~14%) in mean fluorescence intensity between the first and second image acquistions ($p = 0.006$). However, the percentages of mean intensity remaining in the second image acquisition were almost identical between the two groups stored with and without freezing (*$p = 0.992$), indicating that the effect of frozen storage on CF-CHP fluorescence intensity is negligible.

SUPPLEMENTARY TABLE

**Sequence**		**Shear-dominant test 1 (1 N-terminus fixed and 1 C-terminus loaded)**	**Shear-dominant test 2 (2 N-terminus fixed and 1 C-terminus loaded)**
**GEV GPO GPO GPA GEK GSO GAD GPA GSO GTO** (sequence near covalent crosslinking site)	**Peak force (pN)**	621.4	874.7
	**Failure strain (for peak force)**	0.127	0.185
	**Unfolding strain**	0.136	0.16
**GPO GPO GPO GPO GPO GPO GPA GPA GPO GPO** (unnatural hyperstable triple helix sequence)	**Peak force (pN)**	834.9	1201.2
	**Failure strain (for peak force)**	0.235	0.2
	**Unfolding strain**	0.158	0.21

Supplementary Table 1. Summary of simulation results for two different sequences and two different shearing dominant mechanical loading conditions. The sequence near the intermolecular covalent crosslink, which is a thermally unstable sequence (estimated melting temperature: 5.8 °C), has a smaller maximum force and unfolding strain when compared to the unnatural hyperstable sequence (estimated melting temperature: 51.0 °C). The first sequence has a closer interaction with the intermolecular crosslink and thus its results are more relevant to the critical damage strain recorded in our experiments; however, even the hyperstable sequence fails by the shear-dominant mechanism more readily than tension-dominant mechanism suggesting that shear induced failure can take place at almost any triple-helical collagen domain. The relative agreement between the two different shearing conditions suggests that both shear-dominant mechanisms can lead to tension induced damage.

SUPPLEMENTARY METHODS

Data analysis for changes in materials properties of tendon fascicle. The force data for the single stretch experiments were resampled using the 1-D linear interpolation function in Matlab (The MathWorks, Inc.) so that the force data for each sample corresponded to the same strain points. The stress for each sample was calculated assuming a circular cross-section and using the average sample diameter as measured at 5 points along the fascicle in a bright field image. The modulus at each strain point was calculated as the linear modulus using the stress at ±0.0.075% with respect to each strain. The modulus at each strain level used for the single stretch experiment was compared to the maximum modulus (at 2.5% strain) using t-tests with Holm-Sidak correction for multiple comparisons.

For the cyclic experiments, stress was calculated using the average circular cross section area for the single stretch fascicles, as bright field images of the cyclic loaded samples were not available. Modulus as a function of loading cycle was calculated as the linear modulus during loading, between 2-4% strain. Moduli from subsequent loading cycles were compared to the modulus from the first loading cycle using paired t-tests with Holm-Sidak correction for multiple comparisons.

Trypsin digestion and hydroxyproline assay. In preparation for trypsin digestion, rat tail tendon fascicles were strained to different levels (0, 5, 7.5, 9, 10.5, 15%, n = 5) and only the stretched regions between clamps were dissected and used in this assay. To determine wet mass, the approximately 2.5 cm-long dissected fascicles were blotted dry on kimwipe paper and weighed on an analytical balance while immersed in a vial containing 0.01 M Tris-HCl buffer with 20 mM of $CaCl_2$ (Tris/Ca buffer). Each weighed fascicle sample was placed in a polypropylene hydrolysis vial (Sigma 27435) containing 200 µL of Tris/Ca buffer. Using a concentrated stock solution of acetylated trypsin (50 mg mL^{-1}, Sigma T6763) in Tris/Ca buffer, 0.365 mg of acetylated trypsin per mg of wet fascicle was added to each hydrolysis vial. The samples were left to digest under gentle agitation at 22 °C for 24 h.

After trypsin digestion, the percentage of collagen digested by trypsin from each fascicle was assessed using a hydroxyproline assay. From each vial, the 200 µL of digestion solution was completely transferred into a new hydrolysis vial using a long gel-loading tip. Additional 200 µL of Tris/Ca buffer was added to the original hydrolysis vial containing the remaining undigested fascicle. To fully hydrolyze the samples, 200 µL of 12 N HCl (Sigma 320331) was mixed into each hydrolysis vial, and the tightly capped vials were heated in an oven for 18 h at 110 °C. After hydrolysis, the total weight of each vial containing the hydrolyzed solution was measured and compared to a standard vial filled with 400 µL of 1:1 mixture of 12 N HCl and Tris/Ca buffer (mixture-A) to determine the solution loss due to inevitable evaporation during heating. To ensure all vials contain 400 µL of solution, a calculated volume of mixture-A was added to every vial to compensate the evaporated solution. After this calibration step, an aliquot of hydrolysis solution from each vial was used for hydroxyproline quantification. From each vial that contains trypsin-digested collagen, 100 µL of the hydrolysis solution was withdrawn; from each vial that contains the remaining fascicle after trypsin digestion, only 3 µL of the hydrolysis solution was taken and further mixed with 97 µL of mixture-A. Because the concentration of hydroxyproline in the hydrolysis solution from the trypsin-digested portion is much lower than the undigested portion, we found that it is important to use 20-50 folds more hydrolysis solution from the digested portion to ensure all measured hydroxyproline amounts are in the working range of the assay. A standard curve was made using solutions containing hydroxyproline

ranging from 0 to 1 μg in 100 μL of mixture-A. The acidic hydrolysis solution was evaporated to dry under vacuum for over 2 h in microtubes. Water (100 μL) was added to each dried sample aliquot, followed by chloramine-T (100 μL, Sigma MAK008). After 20 min incubation at room temperature, a mixture of perchloric acid and DMAB (100 μL) was added and the entire mixture was heated at 60 °C for 20 min. We found that it is important to add the hydroxyproline assay reagents to the paired vials containing the trypsin-digested and undigested parts of each fascicle in immediate succession to reduce statistical errors that may come from different duration of reaction time for color development. Following color development, the absorbance of a 200 μL aliquot from each colorimetric reaction mixture were read in a 96-well plate at 561 nm using a SpectraMax Gemini XPS microplate reader (Molecular Devices). The percentage of collagen digested by trypsin from each fascicle sample was calculated by dividing the total hydroxyproline content of the digestion solution by the total hydroxyproline content of its undigested portion plus digestion solution.

Following the original methods reported in literature[1,2], our assay produced considerable statistical errors which made it not suitable to detect small amounts of unfolded collagen in these fascicles that were not heavily strained. To solve this problem, we added and modified multiple steps to minimize errors, as described in the underlined sentences above. Results from two digestion assays using the methods before and after improvement are presented in Supplementary Figure 3.

Collagen antibody staining and CHP comparison. Tendon fascicle samples were strained to 0% and 15% (as described in Methods) and incubated in 1 mL of 1×PBS containing 5 μg mL^{-1} of anti-collagen type-I antibody (Abcam ab34710) at 4 °C over night. The samples were removed from the antibody solution and washed three times in 1×PBS for 10 min at room temperature. The samples were incubated in the fluorescent secondary antibody solution (Alexa Fluor 555 labeled Donkey Anti-Rabbit IgG H&L, Abcam ab150074, 6.7 μg mL^{-1}) at room temperature for 1 h before washing three times in 1× PBS and imaging using a Nikon automated widefield fluorescence microscope (Supplementary Figure 13).

Effect of frozen storage on CF-CHP fluorescence intensity. Tendon fascicle samples were stretched to 12% strain (as described in the methods for single stretch experiments) and incubated overnight in 500 μl of 7.5 μM CF-CHP at 4 °C (n = 8). Following incubation, the samples were washed three times in 1 ml of 1×PBS for 30 min at room temperature to remove unbound CF-CHP. All samples were imaged under a widefield fluorescence microscope (as described in the methods for fluorescence imaging and analysis) immediately following the CF-CHP staining and washing procedure. Following imaging, four samples were stored at 4 °C and the remaining four samples were stored at -20 °C overnight and reimaged under the widefield fluorescence microscope the following day. Paired t-tests were used to compare the intensity of all samples between the first and second image acquisition and to compare the second image intensity as a percentage of the first image intensity for the 4 °C and -20 °C storage groups (Supplementary Figure 14). The Holm-Sidak method was used to correct for multiple comparisons.

SUPPLEMENTARY REFERENCE

1. Veres, S. P., Harrison, J. M. & Lee, J. M. Mechanically overloading collagen fibrils uncoils collagen molecules, placing them in a stable, denatured state. *Matrix Biol.* **33,** 54-59 (2014).

2. Willett, T. L., Labow, R. S., Avery, N. C. & Lee, J. M. Increased proteolysis of collagen in an in vitro tensile overload tendon model. *Ann. Biomed. Eng.* **35,** 1961-1972 (2007).
